# HIV-1 Activates T Cell Signaling Independently of Antigen to Drive Viral Spread

**DOI:** 10.1016/j.celrep.2016.12.057

**Published:** 2017-01-24

**Authors:** Alice C.L. Len, Shimona Starling, Maitreyi Shivkumar, Clare Jolly

**Affiliations:** 1 Division of Infection and Immunity, University College London, London WC1E 6BT, UK

**Keywords:** HIV, T cell, signaling, TCR, phosphoproteomics, synapse

## Abstract

HIV-1 spreads between CD4 T cells most efficiently through virus-induced cell-cell contacts. To test whether this process potentiates viral spread by activating signaling pathways, we developed an approach to analyze the phosphoproteome in infected and uninfected mixed-population T cells using differential metabolic labeling and mass spectrometry. We discovered HIV-1-induced activation of signaling networks during viral spread encompassing over 200 cellular proteins. Strikingly, pathways downstream of the T cell receptor were the most significantly activated, despite the absence of canonical antigen-dependent stimulation. The importance of this pathway was demonstrated by the depletion of proteins, and we show that HIV-1 Env-mediated cell-cell contact, the T cell receptor, and the Src kinase Lck were essential for signaling-dependent enhancement of viral dissemination. This study demonstrates that manipulation of signaling at immune cell contacts by HIV-1 is essential for promoting virus replication and defines a paradigm for antigen-independent T cell signaling.

## Highlights

• Unbiased global analysis of T cell signaling changes during HIV-1 cell-cell spread
• Experimental system to map dynamic signaling changes in mixed cell populations over time
• More than 200 host cell proteins are modified as HIV-1 disseminates between T cells
• HIV-1 activates antigen-independent TCR signaling to drive viral spread

## Graphical Abstract

**Figure:**
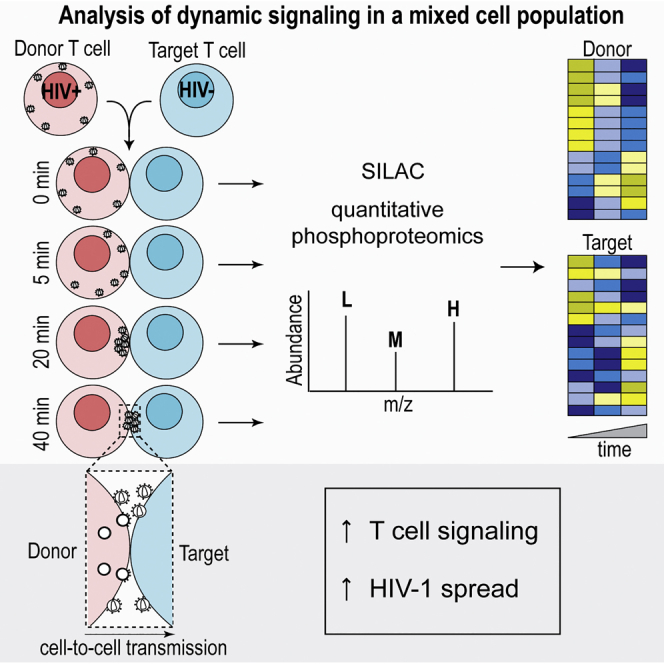

## Teaser

HIV-1 rapidly spreads between T cells. Len et al. have developed an approach to interrogate real-time signaling changes in infected and uninfected cells during viral dissemination. They report that HIV-1-induced cell-cell contact activates antigen-independent T cell signaling that is necessary for HIV-1 to spread efficiently between cells.

## Introduction

Many viruses exploit direct cell-cell infection to replicate most efficiently. HIV-1 is no exception and has evolved to take advantage of the frequent interactions between immune cells in lymphoid tissue to disseminate at sites of T cell-T cell contact (Jolly et al., 2004, Murooka et al., 2012, Sewald et al., 2012). Indeed, cell-cell spread is the predominant mode of HIV-1 replication (Hübner et al., 2009, Jolly et al., 2007b, Martin et al., 2010, Sourisseau et al., 2007) that ultimately leads to T cell depletion and the development of AIDS. HIV-1 manipulation of immune cell interactions in lymphoid tissue, where T cells are densely packed, allows for rapid HIV-1 spread and evasion of host defenses, including innate (Jolly et al., 2010) and adaptive immunity (Malbec et al., 2013, McCoy et al., 2014) as well as antiretrovirals (Agosto et al., 2014, Sigal et al., 2011, Titanji et al., 2013). Importantly, ongoing viral replication likely prevents an HIV/AIDS cure. Cell-cell spread of HIV-1 occurs across virus-induced T cell-T cell contacts (virological synapses [VSs]; Jolly et al., 2004) and is a dynamic, calcium-dependent process that appears highly regulated (Martin et al., 2010, Groppelli et al., 2015), culminating in polarized viral egress and rapid infection of neighboring cells. The molecular details of how HIV-1 co-opts the host cell machinery to drive maximally efficient spread between permissive T cells remains unclear. Moreover, whether cell-cell spread induces signals that potentiate viral replication has been little considered but has major implications for therapeutic and eradication strategies.

Phosphorylation-mediated signaling controls many cellular functions, including immune cell interactions and cellular responses to the environment and infection. Quantitative phosphoproteomics analysis by mass spectrometry (MS) allows for global, in-depth profiling of protein phosphorylation kinetics (Olsen et al., 2006). When coupled with functional analysis, such studies have helped define the pathways leading to T cell activation, differentiation, and gain of effector function, paving the way to understanding the molecular details of T cell signaling and the immune response (Mayya et al., 2009, Navarro et al., 2011, Salomon et al., 2003). So far, analysis of signaling during immune cell interactions has generally employed reductionist approaches; for example, cross-linking individual cell-surface proteins such as the T cell receptor (TCR) or co-stimulatory molecules with antibody (Matsumoto et al., 2009, Mayya et al., 2009, Navarro et al., 2011, Ruperez et al., 2012). Such approaches mimic the process of antigen-dependent stimulation that occurs when a T cell encounters antigen-presenting cells (APCs) expressing cognate peptide in the context of major histocompatibility complex (MHC) molecules. However, the unmet challenge is to globally map cellular signaling pathways activated when two cells physically interact, a more complex setting that recapitulates the uncharacterized complexity of receptor interactions that take place between immune cells and synergize to drive a cellular response.

To gain insight into the molecular mechanisms underlying HIV-1 spread between T cells, we developed an approach that employs triple SILAC (stable isotype labeling by amino acids in cell culture) with quantitative phosphoproteomics to map cellular signaling events simultaneously in two distinct cell populations. We have used this strategy to perform an unbiased and comprehensive analysis of how HIV-1 manipulates signaling when spreading between CD4 T cells. By simultaneously mapping real-time phosphorylation changes in HIV-1-infected and HIV-1-uninfected CD4 T cells with kinetic resolution, we identified the host cell pathways and cellular factors modified during HIV-1 dissemination. Remarkably, our results reveal that HIV-1 subverts canonical TCR signaling in the absence of antigen to drive spread at T cell-T cell contacts. Manipulation of T cell signaling by HIV-1 in this way represents a previously unknown strategy to promote efficient replication with important implications for disease pathogenesis.

## Results

### Widespread Global Signaling Changes Induced during HIV-1 Spread between T Cells

**Figure 1:**
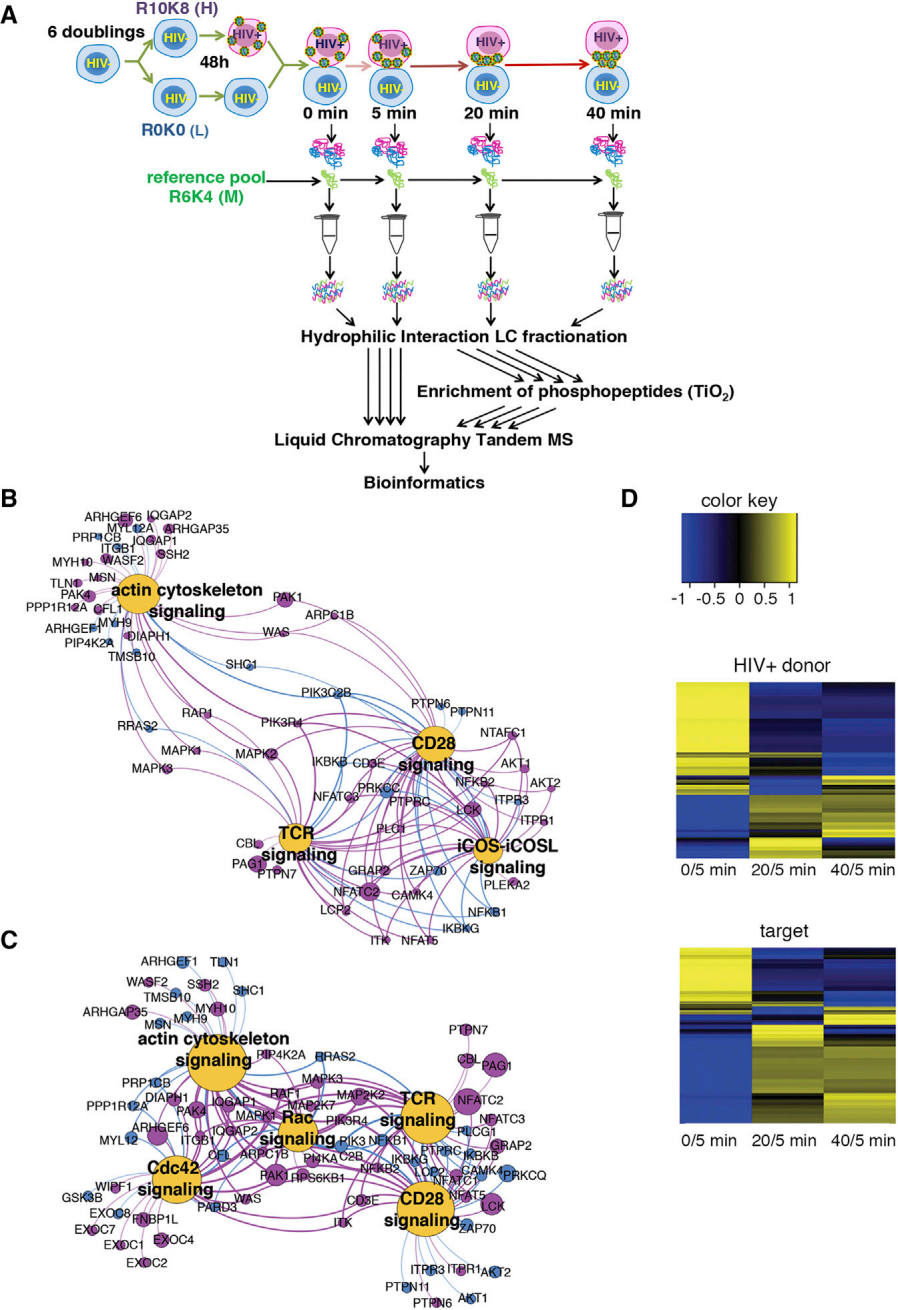
Summary of Phosphoproteome Data (A) Schematic representation of the screen, work flow, and analysis. (B) Top canonical pathways modified in the HIV-1-infected donor T cell from Ingenuity Pathway Analysis (IPA) of experiment 1. Magenta circles indicate proteins with differentially phosphorylated phosphosites (>1.5-fold over time). Blue circles indicate proteins that were identified, but not differentially phosphorylated, in our study. Size of circles represents the magnitude of phosphorylation change. (C) Top canonical pathways modified in the target T cell from IPA. (D) Heatmap depicting quantified phosphorylation sites that demonstrated change in phosphorylation over time from experiment 1. Yellow color denotes increased phosphorylation and blue decreased phosphorylation. Heatmaps were generated using GProX. See also Figures S1–S3 and Tables S1, S2, and S3.

To obtain an unbiased and global overview of manipulation of host cell signaling during HIV-1 spread, we used SILAC coupled with quantitative phosphoproteomics analysis by MS. Jurkat CD4 T cells, a well-characterized model of HIV-1 infection and T cell signaling (Abraham and Weiss, 2004), were labeled using either “heavy” (R10K8) or “light” (R0K0) amino acids for at least six doublings. SILAC-labeled R10K8 T cells were infected with HIV-1 by spinoculation to synchronize infection, achieving 90% infection after 48 hr (Figure S1A). HIV-1-infected heavy- labeled and uninfected light-labeled target T cells were mixed to optimize contacts (see Supplemental Experimental Procedures) and either lysed immediately (0 min) or incubated at 37°C for 5, 20, or 40 min prior to lysis to allow for cell-cell contact and cross-talk (Figures 1A and S1A). We expected rapid dynamics of cellular signaling and HIV-1 cell-cell spread during T cell-T cell contact (Groppelli et al., 2015, Hübner et al., 2009, Jolly et al., 2004). To enable inter-time-point comparison and temporal analysis of dynamic signaling, each time point was supplemented post-lysis with an internal standard consisting of a pooled sample of mixed infected and uninfected T cells both labeled with “medium” (R6K4) amino acids and collected from each time point (Figure 1A). All samples were processed and analyzed by MS with quantification of abundance changes based on MS signal intensities of the triple-SILAC-labeled peptides. Raw MS data were processed using MaxQuant for protein assignment, quantification of peptides, phosphorylation, and phosphosite localization.

We identified a total of 28,853 phosphopeptides corresponding to 5,649 independent proteins (Figures S1A and S1B; Table S1). This is the largest single dataset from a lymphocyte or hematopoietic cell analysis. We captured proteins across numerous subcellular localizations, including the cytoplasm (34.9%), nucleus (47.0%), and plasma membrane (6.2%), in both T cell populations (Figure S1C). Protein function analysis revealed a broad spectrum of host cell pathways modified in both infected and uninfected cells, demonstrating this approach yields an unbiased capture of the T cell phosphoproteome (Figure S1D). Phosphorylated serine and threonine were significantly more abundant than phosphorylated tyrosine (pS/pT/pY = 23,073:6,238:502), in agreement with their relative prevalence and key role in T cell signaling (Mayya et al., 2009, Navarro and Cantrell, 2014, Ruperez et al., 2012).

Co-culturing HIV-1-infected (donor) and uninfected T cells (target) results in >80% of uninfected target T cells becoming infected by contact-dependent cell-cell spread (Martin et al., 2010, Sourisseau et al., 2007). To determine the temporal changes in cellular signaling during HIV-1 spread from donor to target T cells, we curated the data to consider only high-confidence phosphorylation sites (phosphorylation score of >0.75), proteins that were identified at all four time points (0, 5, 20, and 40 min) and those showing >1.5-fold change in the abundance of phosphorylation compared to the internal medium-labeled reference (Table S2). The relative abundance of phosphorylation states for all phosphosites was quantified and the change over time calculated (Table S2). Statistically significant changes over time were detected in 938 phosphopeptides corresponding to 434 proteins in HIV-1 donor cells and 851 phosphopeptides corresponding to 430 proteins in target cells (Figure S1A; Tables S2-2 and S2-7). Consistent with rapid activation of signaling pathways, the largest changes in distribution and frequency of phosphopeptides from both cell populations were seen within the first 5 min (Figures 1D and S2A–S2D). Temporal signaling changes defined groups of early- and late-responsive phosphosites and distinct clusters of responsive proteins, indicative of activation of specific cellular pathways in each cell population and downstream propagation of signaling cascades (Figures 1D, S2E, and S2F). To confirm the data and obviate potential label bias, we repeated the experiment with reversed SILAC labeling. Phosphorylation changes were confirmed in 163 phosphopeptides corresponding to 134 proteins in the HIV-1 donor cell (134/434) (Tables S2-4 and S2-5) and 141 phosphopeptides corresponding to 124 proteins in the target cell (124/430) (Tables S2-9 and S2-10). This represents an average 29% overlap between replicate experiments (Table S2; Figure S3), in excellent agreement with the reproducibility of similar screens (Navarro et al., 2011). Of these, 108 phosphorylation sites were unique to infected cells (Table S3-1), 86 phosphorylation sites were unique to the target cell (Table S3-3), and 55 phosphorylation changes were common to both donors and targets (Tables S3-2 and S3-4). This implicates specific host cell factors that may regulate the late steps of viral assembly and budding (donor cell specific), early infection effects (target cell specific) and common factors that may regulate T cell-T cell interactions and VS formation (overlapping).

### Induction of T Cell Receptor Signaling in HIV-1-Infected Cells

We took an unbiased, Ingenuity Pathway Analysis (IPA) approach to analyze the host signaling networks and pathways modified during HIV-1 spread. This revealed that TCR signaling in donor T cells was the top canonical pathway modified over time during HIV-1 spread (TCR p value = 1.24 × 10^−12^). This was followed by CD28 (p value = 4.61 × 10^−11^), inducible T cell costimulator-inducible T cell costimulatory ligand (iCOS-iCOSL) (p value = 5.5 × 10^−9^), and actin cytoskeleton signaling (p value = 1.2 × 10^−8^) (Figure 1B; Table S2). In uninfected target T cells, the top canonical pathways were TCR (p value = 5.39 × 10^−9^), CD28 (p value = 6.04 × 10^−7^), Cdc42 (p value = 8.73 × 10^−7^), RAC (p value = 1.02 × 10^−7^), and actin signaling (p value = 3.34 × 10^−7^) (Figure 1C; Table S2). Motif-X analysis of phosphosites predicted the kinases most active in HIV-1 donor cells as CaMKII, PAK, and proline-directed kinases, compared to PAK, CDK5, and proline-directed kinases in target cells (Figures S1E and S1F).

**Figure 2:**
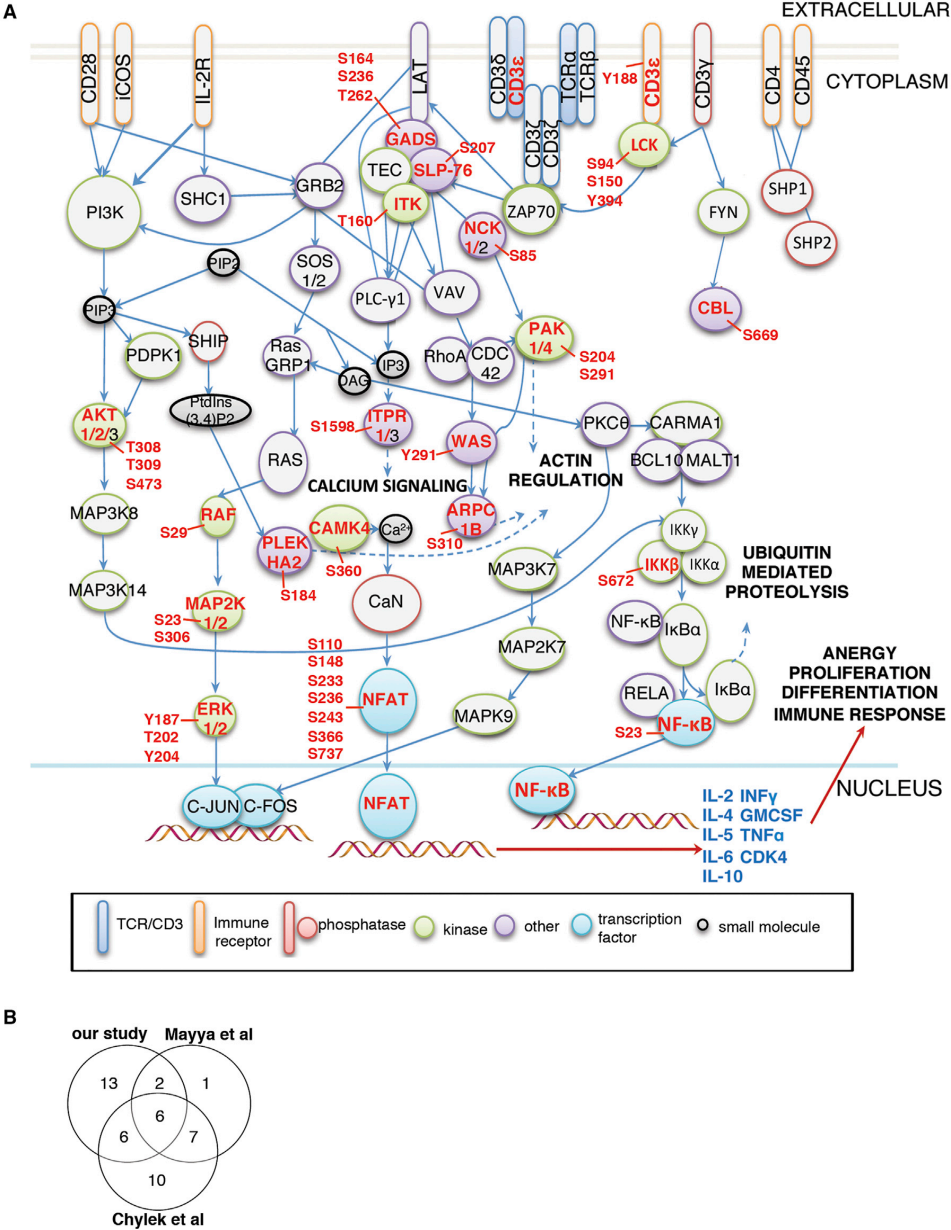
HIV-1-Infected T Cells Show Activation of Immune Receptor Signaling Pathways following Cell-Cell Contact (A) Summary diagram of the TCR, CD28, and iCOS-iCOSL signaling pathways adapted from IPA. Proteins names in red text were identified in our study to be differentially phosphorylated in HIV-1-infected cells in response to cell-cell contact (refer to Tables S1 and S2). For each phosphorylated protein, the specific phosphosite modified is indicated with adjacent red text (e.g., Y187, Y202, and Y204 for ERK). Proteins named in black text map to the TCR, CD28, and iCOS-ICOSL pathways but were either not identified in our study or did not show phosphorylation changes. Colored outlines and shapes of proteins denote protein function (e.g., TCR/CD3, immune receptor, phosphatase, kinase, transcription factor, small molecule, and other). (B) Overlap between phosphosites identified in our study mapping to top canonical pathways modified in HIV-1-infected cells (refer to Table S2) and those identified as TCR responsive by Mayya et al. (Mayya et al., 2009) and Chylek et al. (Chylek et al., 2014). See also Table S4.

The fact that TCR signaling was the most highly activated pathway in infected cells is surprising because HIV-1 mediated T cell-T cell contact during viral spread does not involve TCR-pMHC (peptide major histocompatibility complex) interactions and as such is antigen-independent. Rather it is driven by Env expressed in infected cells engaging viral entry receptors (CD4 and CCR5 or CXCR4) on opposing cells during T cell-T cell interactions with additional contributions from adhesion molecules (LFA-1 and ICAM) (Chen et al., 2007, Jolly et al., 2004, Jolly et al., 2007a, Rudnicka et al., 2009, Sourisseau et al., 2007). Figure 2A graphically summarizes the phosphoproteins we identified in HIV-1 donor cells mapped onto signaling pathways associated with canonical T cell activation at T cell-APC contacts. This visual representation highlights the significant overlap between the well-established TCR/co-stimulatory signaling pathway and phosphorylation changes identified in HIV-1 donor cells during contact with targets.

To explore this further, we compared our phosphoproteome data with studies where the TCR was directly cross-linked on Jurkat T cells, and signaling was analyzed across similar time points. We found a 44% overlap between the phosphorylation profile of HIV-1 donor cells during co-culture with target cells and TCR-specific responses reported by Chylek et al. (Chylek et al., 2014) and a 30% overlap with Mayya et al. (Mayya et al., 2009) (Figure 2B; Table S4-2). KEGG database analysis also reported substantial overlap between our phosphoproteome results and phosphorylation of TCR-associated proteins (Table S4-1).

Interestingly, we identified multiple proteins in our data with phosphorylation changes that mapped to early plasma membrane proximal (CD3, Lck, CD43, CD2AP, GADS, and talin) and intermediate/late components of TCR signaling, as well as downstream regulators of gene expression (ERK1/2, AKT, ETS1, and NFAT) (Tables S1 and S2). Many of the residues modified were known activating sites (Tables S2-5 and S2-10). T cell signaling modulates the host cell cytoskeleton and the protein trafficking that is required for T cell activation and secretion of effector molecules (Brownlie and Zamoyska, 2013, Gomez and Billadeau, 2008). Consistent with the notion that HIV-1 cell-cell spread is an active, cytoskeletal-dependent process and that virus infection drives this process (Jolly et al., 2004), we found dynamic phosphorylation changes to many actin regulators (PAK1, CFL, PALLD, MYH10, VIM, and WAS), polarity proteins (SCRIB) and components of vesicle trafficking and fusion (SNAP23) (Table S2), most of which that have not been previously described as host cofactors for HIV-1 replication and spread.

HIV-1 predominantly spreads at virus-induced cell-cell contacts but can also disseminate less efficiently via classical diffusion-limited cell-free infection. Comparative analysis of our results obtained from target T cells with a study mapping phosphorylation in T cells exposed to cell-free virus (Wojcechowskyj et al., 2013) showed a 23% overlap in modified proteins, with 41% of the phosphorylation changes in these proteins mapping to the same site (Table S5). Since the molecular processes of HIV-1 entry and the early steps of infection are similar between cell-free and cell-cell spread (Jolly et al., 2004, Jolly and Sattentau, 2004), some overlap is expected; however, differences implicate additional signaling pathways specifically activated during T cell-T cell contact and other unique responses occurring when target cells encounter greater numbers of incoming virions during cell-cell spread (Jolly et al., 2004, Martin et al., 2010, Sourisseau et al., 2007).

### TCR Signaling by a Non-canonical Means Employs Classical T Cell Kinases

**Figure 3:**
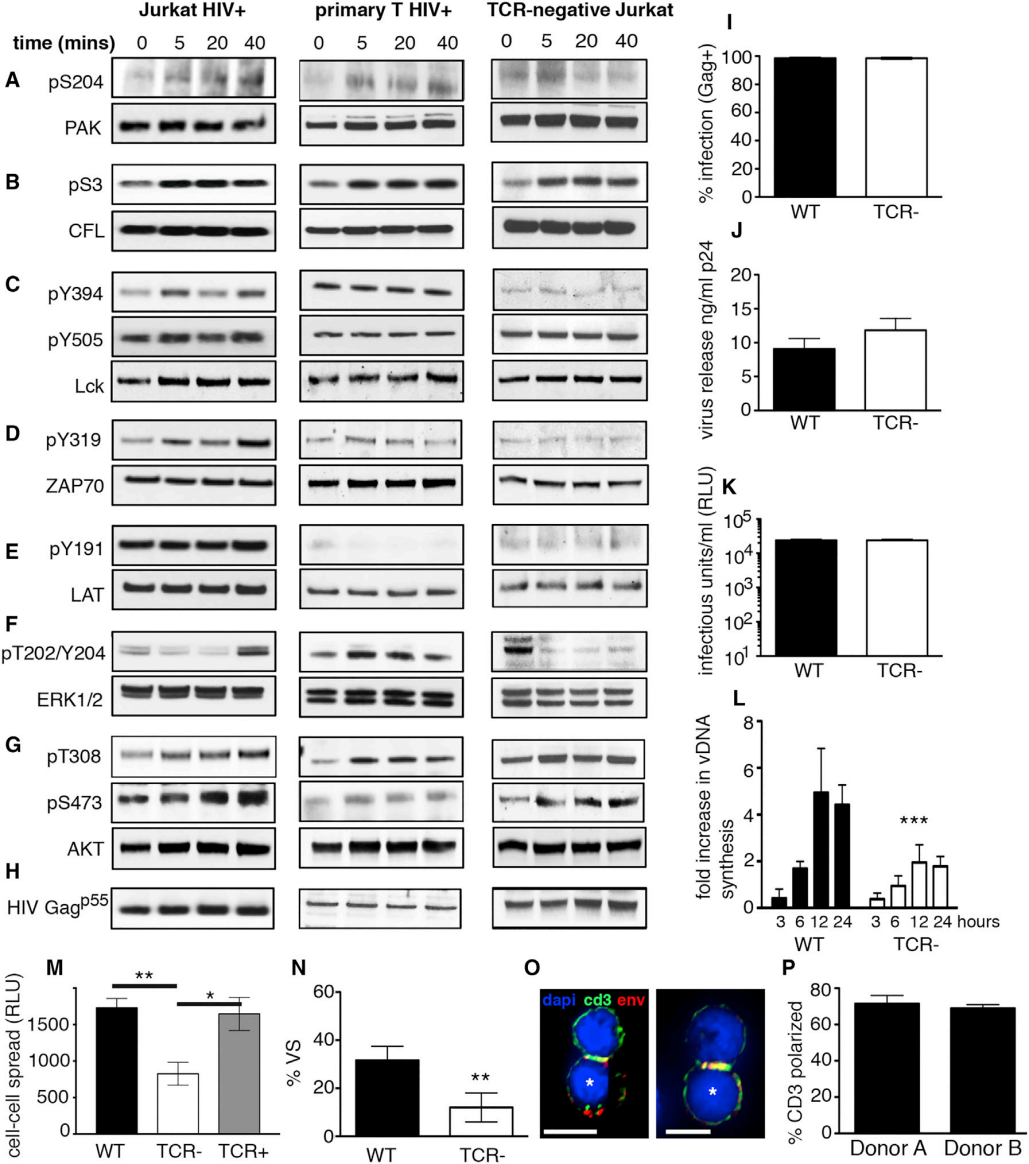
T Cell Receptor Signaling Drives HIV-1 Cell-Cell Spread (A–H) Protein phosphorylation analysis by western blotting of lysates prepared from contacts between infected and uninfected T cells. Blots are representative of at least two independent experiments. Antibodies specific for phosphorylated and total protein were used (A–G). HIV-1 Gag p55 confirms infection and equal loading (H). The left panels show HIV-1 infected Jurkat T cells and uninfected Jurkat targets. The middle panels show HIV-1-infected primary CD4 T cells and uninfected primary CD4 targets. The right panels show HIV-1 infected TCR-negative Jurkat T cells and uninfected WT Jurkat targets. (I–L) Jurkat T cells were infected with VSV-G pseudotyped HIV-1. (I) Infected cells were quantified by flow cytometry. (J) Virus budding was measured by Gag p24 ELISA. (K) Virion infectivity was measured by luciferase assay. (L) Quantification of cell-cell spread by real-time qPCR. Values show the fold-increase in viral DNA over time compared to the baseline (0 hr), reflecting de novo reverse transcription in newly infected target cells. (M) Quantification of cell-cell spread from infected WT, TCR-negative, and TCR-reconstituted cells measured by luciferase assay (RLU, relative light units). (N) HIV-1-infected TCR-negative cells form fewer VSs (number of cell-cell contacts analyzed: WT, n = 29; TCR negative, n = 32). (O) The TCR complex component CD3 is co-polarized with HIV-1 Env at the interface formed between HIV-1-infected primary T cells (bottom, asterisk) and autologous target T cells (top). Scale bar, 5 μm. (P) Quantification of the frequency of CD3 enrichment at the contact zone (number of cell-cell contacts analyzed: donor A, n = 41; donor B, n = 13). Data represent mean ± SEM from two or three independent experiments. ^∗∗^p < 0.005; ^∗∗∗^p < 0.001. See also Figures S4 and S5.

Having identified changes in phosphorylation of key components of classical T cell signaling during HIV-1 spread, which strongly indicates activation, we sought to validate this observation directly using western blotting to visualize protein phosphorylation and quantified this from multiple experiments using densitometry analysis (Figures 3, S4, and S5). Proteins were chosen that represented upstream kinases, cytoskeletal proteins, and transcriptional regulators involved in T cell receptor signaling that showed dynamic phosphorylation changes at defined sites that dictate protein function (e.g., Lck^Y394^, PAK1^S204^, CFL^S3^, ERK^T202/Y204^, AKT^T308^, and AKT^S473^). Other components of the top canonical pathways activated (e.g., ZAP70^Y319^ and LAT^Y191^) were also included. Figures 3A and 3B shows that contact between HIV-1-infected and HIV-1-uninfected T cells increased phosphorylation of the actin regulators PAK1^S204^ and CFL^S3^. While PAK1 activation was specific to contacts mediated by HIV-1-infected cells, CFL phosphorylation appeared to be infection-independent and was also triggered by contact between uninfected T cells (Figures S4 and S5). However, as T cells do not usually form sustained cell-cell contacts in the absence of retroviral infection (Jolly and Sattentau, 2004) or previous antigenic stimulation (Sabatos et al., 2008), this may be unlikely to occur under normal conditions of transient cell interactions. PAK1 influences cytoskeletal dynamics and T cell activation and is activated through phosphorylation at Ser204 via TCR-dependent (Yablonski et al., 1998) and TCR-independent mechanisms (Phee et al., 2005). CFL, a downstream target of the PAK1 cascade, stimulates actin severance and depolymerization to increase actin turnover and is inactivated by LIMK phosphorylation at Ser3 (Yang et al., 1998), potentially stabilizing cell-cell contacts. Modulation of cytoskeletal dynamics is thus consistent with the requirement for actin remodeling during immune cell interactions and HIV-1 cell-cell spread (Jolly et al., 2004, Jolly et al., 2007b, Rudnicka et al., 2009). Next, we examined Lck and ZAP70 (Figures 3C, 3D, S4, and S5), which are TCR-proximal kinases and key initiators of T cell signaling (Brownlie and Zamoyska, 2013, Iwashima et al., 1994). Lck activity is regulated by multiple phosphorylation states (activating site Lck^Y394^; inhibitory site Lck^Y505^) and intracellular localization (Casas et al., 2014, Nika et al., 2010, Salmond et al., 2009). ZAP70 activity is positively regulated by Y319 phosphorylation (Di Bartolo et al., 1999). Consistent with activation of TCR signaling, rapid and dynamic changes to both Lck^Y394^ and ZAP70^Y319^ were seen over time during HIV-1-induced cell-cell contact (Figures 3C, 3D, S4, and S5), with identical patterns of phosphorylation indicative of Lck-dependent ZAP70 activation (Brownlie and Zamoyska, 2013). A slight dip in Lck and ZAP70 phosphorylation was seen at 20 min, although the reasons for this are unclear. By contrast, activation of LAT^Y191^ was unchanged in both MS and western blotting (Figures 3E, S4, and S5; Table S1). Supporting our phosphoproteome data showing downstream propagation of signaling cascades (Figure 2), strong activation of ERK^T202/Y204^ during HIV-mediated cell-cell contact was observed by 40 min (Figures 3F, S4, and S5). Finally, having found phosphorylation of the serine/threonine kinase AKT and a number of downstream targets by MS (e.g., TSC1, TBS1D4, PTPN1, Girdin, GSK3β, and HTT; Table S2), we tested phosphorylation of AKT^T308^ and AKT^S473^. AKT^T308^, which lies in the activation loop of AKT and is most correlative with kinase activity (Alessi et al., 1997), showed a 1.5-fold increase in phosphorylation during HIV-1-mediated cell-cell contact (Figures 3G, S4, and S5). By contrast, AKT^S473^ that contributes to further kinase function (Sarbassov et al., 2005), and phosphorylation of additional downstream targets appeared to be activated by cell-cell contact independent of HIV-1 infection (Figures 3G, S4, and S5).

Next, we extended the analyses to primary CD4 T cells purified from healthy donors that were infected with HIV-1 ex vivo and mixed with autologous CD4 T cells (Figures 3 and S5) as well as mock-infected controls (Figures S4 and S5). Primary T cells showed similar patterns of HIV-dependent, contact-mediated phosphorylation over time but more rapid propagation of signaling and more robust AKT^T308^ activation (Figures 3 and S5), in agreement with previous data indicating HIV-1-infected primary T cells are highly responsive to contact with target cells (Groppelli et al., 2015). However, western blotting of total cell lysates from primary cells did not reveal global changes in Lck phosphorylation (Figures 3C and S5), consistent with high basal levels of Lck phosphorylation in primary T cells (Nika et al., 2010).

### The T Cell Receptor Is Required for Efficient HIV-1 Spread at T Cell Contacts

Signaling through the TCR is considered a tightly controlled checkpoint to ensure T cell activation only occurs in response to foreign antigen displayed by MHC. It is therefore striking that antigen-independent, HIV-1-induced T cell-T cell interactions should trigger classical TCR signaling cascades and phosphorylation of numerous pathway components. To probe the relationship between the TCR complex and contact-induced activation of signaling in HIV-1 donor cells, TCR/CD3-negative T cells were infected with HIV-1 and phosphorylation examined. Notably, HIV-1-infected TCR-negative cells did not phosphorylate PAK, Lck, ZAP70, or ERK in response to contact with target T cells (Figures 3A, 3C, 3D, 3F, and S5), implicating the TCR in signal activation. As a control, we confirmed TCR-negative cells retained expression of Lck (Figure S4L) and that HIV-1-infected cells did not downregulate cell-surface expression of the TCR/CD3 complex (Figures S4J and S4K).

Seeking a role for TCR-dependent signaling in HIV-1 spread, TCR-negative cells were infected and their ability to support viral replication measured. TCR-negative cells were readily susceptible to initial infection with HIV-1 (Figure 3I) and showed no defect in cell-free virus production over a single round of infection, as measured by quantifying release of viral Gag (budding) and particle infectivity (Figures 3J and 3K). Remarkably, when infected cells were incubated with wild-type target T cells, we observed a significant defect in their ability to transmit virus by cell-cell spread (Figure 3L). Reconstituting TCR expression using lentiviral transduction resulted in >85% of cells expressing the TCR complex at the cell surface (Figure S4I) and rescued HIV-1 cell-cell spread (Figure 3M). Failure of TCR-negative cells to efficiently transmit virus by cell-cell spread indicates an important role for the TCR in VS formation and virus spread. Quantitative immunofluorescence microscopy (Figure 3N) revealed TCR-negative cells were indeed impaired in VS formation and could not recruit the viral structural proteins Gag and Env to sites of cell-cell contact to polarize viral budding toward the target cell (Env enrichment at contact site: wild-type (WT), 10-fold ± 2.6-fold, n = 17; TCR negative, 1.6-fold ± 0.5-fold, n = 16; Gag enrichment at contact site: WT, 18.3-fold ± 5.7-fold, n = 14; TCR negative, 1.7-fold ± 0.3-fold, n = 16).

We hypothesized that close and sustained contact between infected and uninfected cells may be inducing TCR coalescence at the contact site as a mechanism of receptor triggering (van der Merwe and Dushek, 2011). In support of this model, analysis of contacts formed between HIV-1-infected primary T cells and autologous uninfected T cells showed that 70% of VSs displayed co-enrichment of the TCR and Env on infected cells at the contact zone (Figures 3O and 3P), despite the absence of direct antigen-dependent TCR engagement by opposing uninfected targets. Quantification of fluorescence revealed the TCR was enriched 3.3-fold ± 0.6-fold and Env 6.9-fold ± 1.5-fold at the contact site (n = 20).

### Lck and ZAP70 Support HIV-1 Spread between T Cells

**Figure 4:**
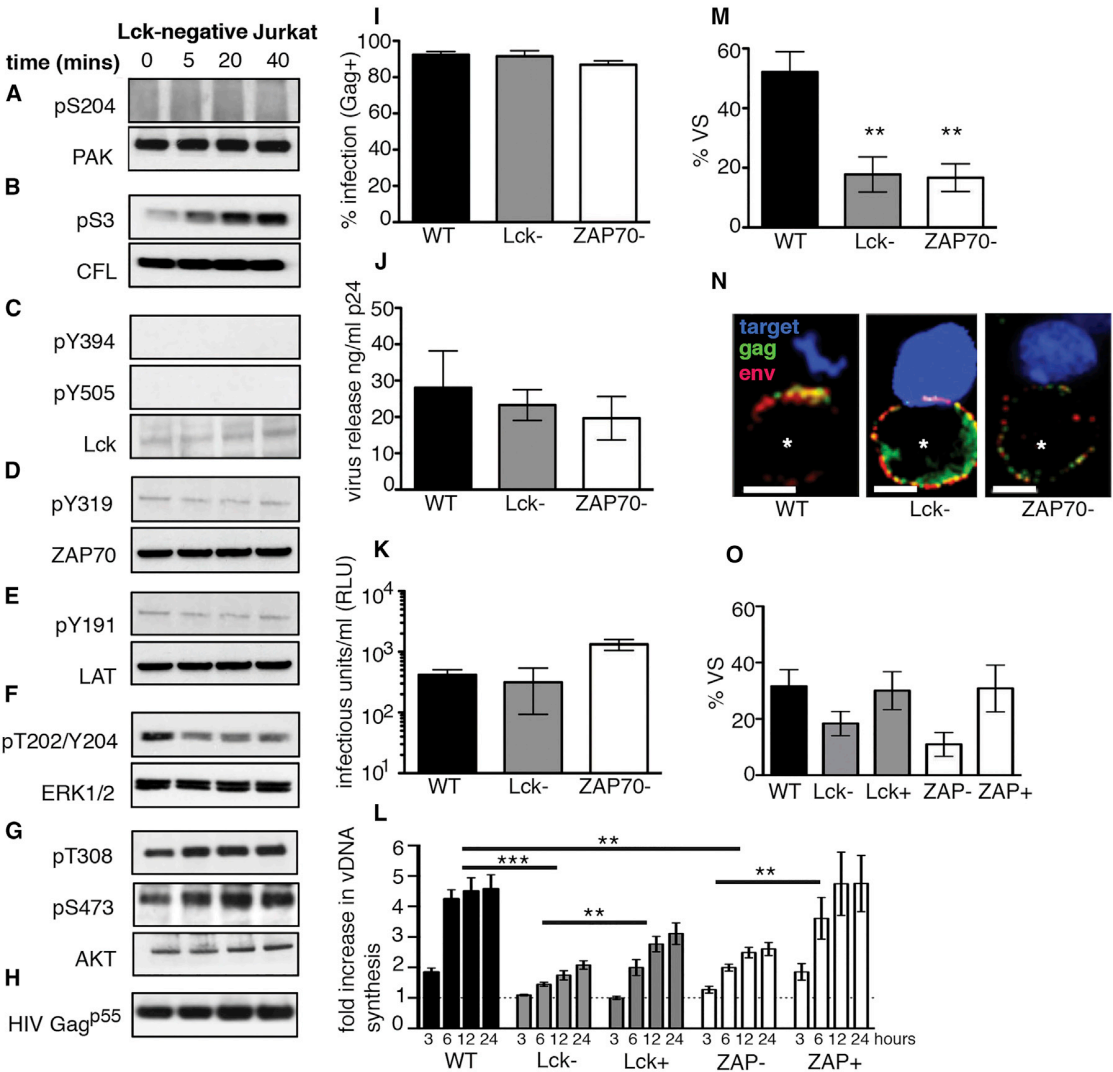
T Cell Kinases Lck and ZAP70 Are Required for Efficient HIV-1 Spread (A–H) Protein phosphorylation analysis by western blotting of lysates (A–G) (see Figure 3) prepared from contacts between HIV-1 infected Lck-negative Jurkat T cells and uninfected wild-type Jurkat targets. Blots are representative of at least two independent experiments. (I) Quantification of infection by flow cytometry. (J) Quantification of cell-free virus budding by Gag p24 ELISA. (K) Particle infectivity determined by reporter cell luciferase assay. (L) Quantification of HIV-1 cell-cell spread from infected Jurkat T cells by real-time qPCR. (M) Percentage of infected Jurkat (Gag, green; Env, red) and uninfected target cells (dye-labeled, blue) contacts showing polarization of Env and Gag to the contact zone (percentage of virological synapses [VSs]) (number of cell-cell contacts analyzed: WT, n = 30; Lck negative, n = 30; ZAP70 negative, n = 30). (N) Representative images of normal VSs (WT) and defective VSs (Lck and ZAP70 negative) formed between an HIV-1-infected Jurkat T cell (bottom, asterisk) and an uninfected target T cell (top). (O) Reconstituting Lck and ZAP70 expression restores VS formation (number of cell-cell contacts analyzed: WT, n = 59; Lck negative, n = 58; Lck positive, n = 48; ZAP70 negative, n = 60; ZAP70 positive, n = 38). Data represent mean ± SEM from three independent experiments. ^∗∗^p < 0.005; ^∗∗∗^p < 0.001. See also Figures S4 and S5.

The kinase Lck is a key upstream initiator of TCR signaling and activation of cytoskeletal dynamics at immune cell contacts (Danielian et al., 1991, Iwashima et al., 1994, Lovatt et al., 2006, Nika et al., 2010, Straus and Weiss, 1992). To test whether signaling during HIV-1-induced T cell contact was Lck dependent, Lck-negative JCAM1.6 cells were infected with virus and mixed with wild-type target T cells, and protein phosphorylation was analyzed. Figures 4A–4G and quantification of western blots (Figure S5) revealed that Lck-negative HIV-1-infected cells were unable to initiate signaling and activate PAK1^S204^, ZAP70^Y319^, ERK^T202/Y204^, and AKT^T308^, whereas CFL remained responsive. To examine whether Lck and the downstream kinase ZAP70 contribute functionally to HIV-1 replication, Lck- and ZAP70-negative T cells were infected, and virus assembly, budding, and spread were quantified. We used VSV-G-pseudotyped virus to overcome variable expression of the receptor CD4. Notably, both Lck- and ZAP70-negative Jurkat cells failed to support efficient cell-cell spread (Figure 4L). In agreement with data using TCR-defective cells, impaired cell-cell spread in Lck- and ZAP70-deficient Jurkat cells was not due to a block in virus infection or a defect in virus production, since the cell-free virus budding and particle infectivity were equivalent to that of WT Jurkat cells (Figures 4I and 4K). However, as expected, there was a significant defect in VS formation and failure to recruit Env and Gag to the contact interface (Figures 4M and 4N) but no effect on cell-cell contact (WT, 27%; Lck negative, 20%; and ZAP70 negative, 19%; p > 0.05), demonstrating that Lck and ZAP70 are not mediating their effect through altering T cell-T cell interactions. Reconstituting cells with exogenous Lck and ZAP70 (Figures S4M and S4N) significantly increased cell-cell spread (Figure 4l) and restored VS formation (Figure 4O) as measured by Env and Gag recruitment to the cell-cell interface (Env enrichment at contact site: Lck negative, 2.6-fold ± 1.5-fold, n = 15; Lck reconstituted, 9.5-fold ± 4.5-fold, n = 15; ZAP70 negative, 3.1-fold ± 1.2-fold, n = 14; ZAP70 reconstituted, 10.7-fold ± 3.6-fold, n = 12; Gag enrichment at contact site: Lck negative, 1.9-fold ± 0.8-fold, n = 15; Lck reconstituted, 5.6-fold ± 1.8-fold, n = 17; ZAP70 negative, 2.4-fold ± 0.7-fold, n = 16; ZAP70 reconstituted, 14.5-fold ± 6.6-fold, n = 12).

### Viral Determinants of Contact-Induced T Cell Signaling

**Figure 5:**
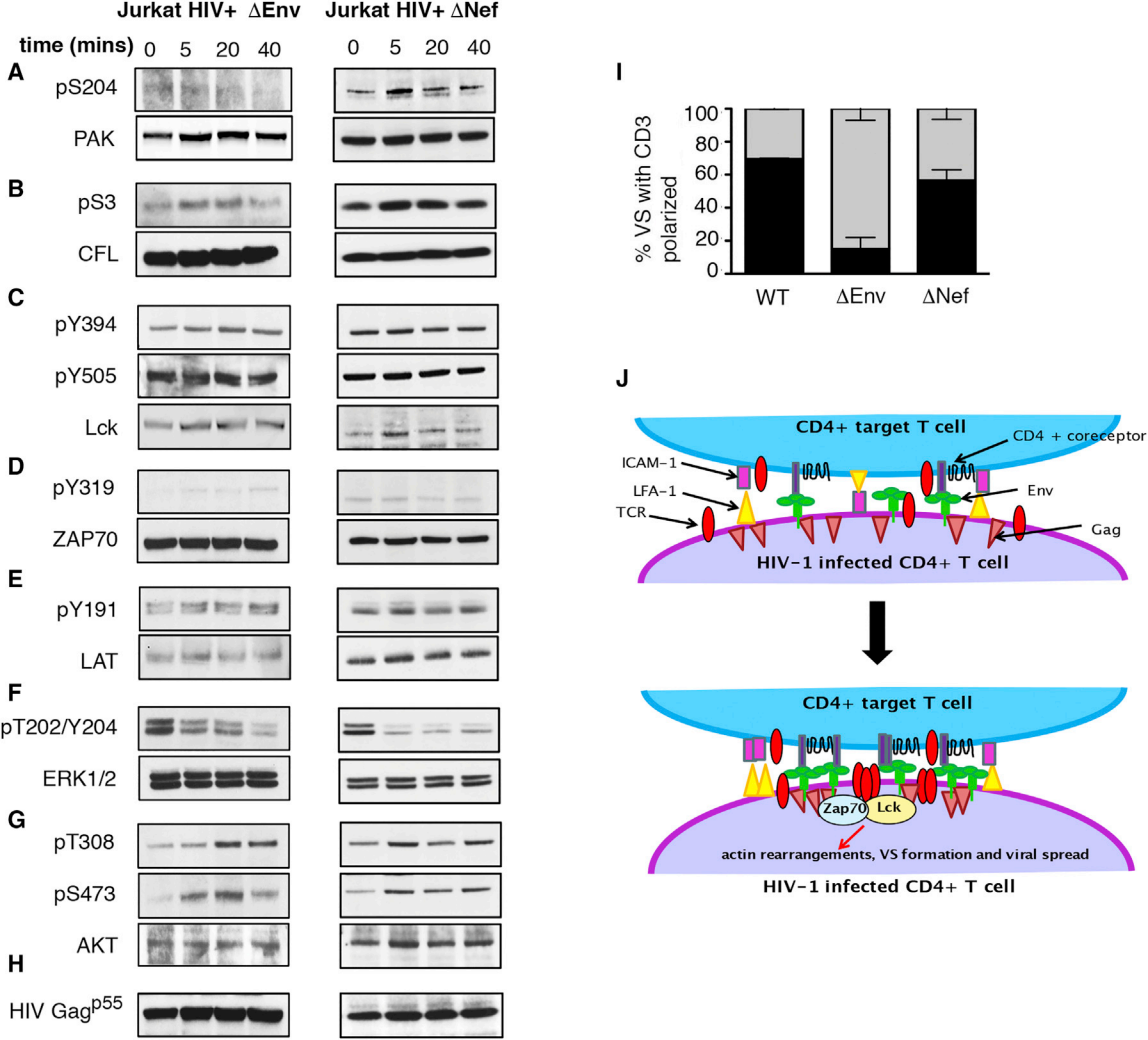
Viral Determinants of Contact-Induced TCR Signaling (A–H) Protein phosphorylation was determined by western blotting (A–G) (see Figure 3). Blots are representative of at least two independent experiments. The left panels show Jurkat T cells infected with VSV-G-pseudotyped HIV-1 containing a frameshift mutation in Env (ΔEnv) and incubated with uninfected target cells. The right panels show Jurkat T cells infected with VSV-G-pseudotyped ΔNef HIV-1 and incubated with uninfected target cells. (I) Quantification of the frequency of CD3 enrichment at the contact zone using viral mutants (black filled, percentage of CD3-enriched contacts; gray filled, percentage of CD3-nonenriched contacts) (number of cell-cell contacts analyzed: WT, n = 54; ΔEnv, n = 31; ΔNef, n = 30). Data from two independent primary T cell donors with the SEM. (J) Model depicting contact-induced activation of TCR signaling during HIV-1 cell-cell spread. See also Figures S4 and S5.

The viral determinants of contact-induced antigen-independent T cell signaling remained unclear. HIV-1 Env expressed on the surface of infected T cells binds cellular entry receptors on opposing cells, leading to sustained cell-cell contact, plasma membrane remodeling, receptor clustering, and VS formation (Jolly et al., 2004). Consistent with antigen-independent T cell signaling being driven by close, sustained physical cell contact mediated by Env-CD4/coreceptor interactions, T cells infected with Env-deleted VSV-G-pseudotyped virus (HIV+ ΔEnv) did not activate phosphorylation of T cell signaling components following incubation with target cells, with the exception of CFL and AKT^473^ (Figures 5A–5F and S5). Similar results were observed when primary CD4 T cells were infected with ΔEnv VSV-G-pseudotyped virus (Figures S4 and S5). We postulated that failure to activate signaling was because TCR clustering did not occur in the absence of HIV-1 Env-mediated cell-cell contact. Concordantly, we observed a significant reduction in the number of cell-cell contacts displaying TCR clustering in the absence of HIV-1 Env expression on infected primary CD4 T cells (Figure 5I), with only 16% of contacts showing TCR enrichment when cells were infected with HIV ΔEnv virus compared to 70% using WT virus (p < 0.001).

The HIV-1 accessory protein Nef has been reported to modulate T cell signaling (Pan et al., 2012, Simmons et al., 2001) and induce hyper-responsiveness to stimulation (Hanna et al., 1998, Schrager and Marsh, 1999, Wang et al., 2000). To test whether Nef was potentiating antigen-independent signaling, T cells were infected with Nef-deleted virus and signaling examined. Figures 5A–5G shows that deletion of Nef resulted in failure to activate ERK^T202/Y204^, Lck^Y394^, ZAP70^Y319^, and PAK1^S204^ following incubation with target cells, with AKT^T308^ phosphorylation remaining responsive to cell-cell contact (Figures 5 and S5). However, in contrast to Env, Nef appeared dispensable for TCR clustering at the VS (Figure 5I), suggesting Nef potentiation of signaling acts downstream of cell-cell contact. Taken together, these data demonstrate HIV-1 infection induces antigen-independent TCR signaling that is activated by Env-dependent cell-cell contact and further potentiated by the HIV-1 virulence factor Nef.

## Discussion

Here, we have developed an approach to globally map phosphorylation-based dynamic signaling events in complex mixed cell populations and performed an analysis of host cell signaling pathways that are manipulated during HIV-1 spread between CD4 T cells. Cell-cell spread of HIV-1 potently enhances viral dissemination (Hübner et al., 2009, Jolly et al., 2007b, Martin et al., 2010, Sourisseau et al., 2007), but many aspects of this host-pathogen interaction remain obscure. Our identification of >200 host cell factors that are manipulated during highly efficient HIV-1 spread is thus timely and provides a wealth of information with implications for pathogenesis. Furthermore, the experimental approach we describe has broad applicability and can be readily applied to complex biological analyses such as signaling during intercellular communication or to define host cell responses to the sequential steps of pathogen replication.

Notably, we make the unexpected discovery that HIV-1 subverts classical TCR signaling pathways independently of antigen during cell-cell contact to drive viral spread from infected to uninfected T cells. Specifically, we found that cell-cell contact mediated by Env uniquely activates the TCR/CD3 complex and downstream kinases Lck and ZAP70 in infected T cells and that this process is required to transmit virus to neighboring cells. We propose a paradigm (model) of TCR signaling (Figure 5J) in which the close apposition and sustained physical contact between HIV-1-infected and -uninfected T cells, which are mediated by Env-receptor interactions and remodel the synaptic plasma membrane, lead to aggregation and enrichment of the TCR/CD3 complex at the contact site and initiation of TCR signaling. Specifically, the presence of plasma-membrane-exposed Env engaging CD4 and coreceptor on target cells results in coalescence and enrichment of cross-linked Env at the VS (Figures 3 and 4) (Jolly et al., 2004). Concomitantly, we observe Env-dependent clustering of TCR-containing plasma membrane microdomains at the cell-cell interface (Figures 3 and 5) and activation of Lck-dependent signaling. We propose that activating signaling could then drive the recruitment of additional Env and Gag to the contact zone as we found here by quantifying viral protein enrichment (potentially via cytoskeletal remodeling and activation of intracellular transport pathways), resulting in polarized HIV-1 assembly and budding of virions across the synaptic space toward the engaged target cell. The TCR, Lck and ZAP70 comprise a triad of early T cell signaling initiators that trigger cytoskeletal remodeling and intracellular trafficking. Consistent with this, these host proteins were all required to direct the active transport of HIV-1 structural proteins Env and Gag to sites of cell-cell contact. In support of our results, ZAP70 has previously been implicated in HIV-1 spread and VS formation (Sol-Foulon et al., 2007). While our data reveal a compelling role for antigen-independent TCR triggering for synaptic signaling, VS formation, and rapid HIV-1 transmission, we cannot discount the contribution of other cell-surface receptors, such as LFA-1 (which is recruited to both the immunological and virological synapses) or indeed other as-yet-unidentified pathways, in contact-induced signaling during HIV-1 spread. Unfortunately, ablation of LFA-1 expression significantly impaired the stability of cell-cell contacts (data not shown), meaning we were unable to assess the contribution of adhesion molecules to signaling. Future work will undoubtedly be informative to further define these processes.

Jurkat cells have been used extensively to interrogate T cell signaling and HIV-1 cell-cell spread; however, they are constitutively activated and can lack components of the T cell signaling machinery. For example, PTEN deficiency results in higher levels of basal AKT activation in transformed cell lines, including Jurkats (Xu et al., 2002). This is reflected in our results, where we detected more robust contact-dependent AKT^T308^ phosphorylation in primary T cells compared to Jurkat cells. By contrast, primary T cells show high basal Lck activation (Nika et al., 2010), making it difficult to detect differential Lck phosphorylation by western blotting, as we also observed. However, having performed experiments using both Jurkats and primary T cells, we provide compelling data attesting to contact-mediated activation of T cell signaling that is dependent on HIV-1 infection and Env expression. This was supported by our observation that the TCR complex is recruited to the VS formed between primary T cells in an Env-dependent manner. Furthermore, cell lines lacking the TCR or Lck were unable to activate signaling, form VSs, and drive viral spread. That we were previously unable to detect significant enrichment of CD3 at the VS in an earlier study using T cell lines (Jolly et al., 2004) is likely to due to suboptimal staining conditions and the choice of cells in that study. Here, we find that cell permeabilization prior to staining with commercial antibody and the use of primary CD4 T cells greatly improved the intensity of CD3 staining and revealed robust and reproducible enrichment of TCR/CD3 at the VS.

Our simultaneous analysis of phosphorylation changes in HIV-1-infected and HIV-1-uninfected T cells during viral dissemination revealed widespread modulation of host cell pathways by HIV-1 that support viral replication by activating unique replication-enhancing signals. In addition to the requirement for physical cell-cell contact mediated by Env, the viral accessory protein Nef was necessary, but not sufficient, for contact-induced TCR signaling. Nef is multifunctional modulator of T cell signaling (Arhel and Kirchhoff, 2009) that has been implicated in aberrant Lck activation independent of the TCR during T cell-APC interactions and perturbation of immune synapse formation (Pan et al., 2012, Thoulouze et al., 2006). However, conflicting reports about Nef’s ability to potentiate or suppress signaling means the biological consequences for viral spread remain poorly understood. Intriguingly, the Nef proteins of HIV-1 and its ancestor, SIV_cpz_, unlike most other simian immunodeficiency viruses (SIVs), do not downregulate expression of the TCR on infected T cells (Arhel et al., 2009, Schindler et al., 2006). Why HIV-1 does not employ this potential immune-evasion strategy has remained enigmatic. We propose that HIV-1 has instead evolved to preserve expression and exploit the TCR through molecular reprogramming of classical T cell signaling pathways during cell-cell contact, allowing for optimal viral replication and spread between human CD4 T cells. That most SIVs downregulate the TCR on infected cells raises the intriguing question of how those viruses disseminate between cells. Whether they exploit an alternate mechanism for driving spread between T cells in contact, and if this contributes to differences in immune activation and disease pathogenesis seen in natural SIV infections (Kirchhoff, 2009), is unknown. Future studies to address this would be extremely interesting and undoubtedly shed new light on unresolved questions surrounding the pathogenicity of lentiviral infection.

We envisage that the insights our data provide into the manipulation of T cell signaling by HIV-1, coupled with the identification of >200 host cell factors modified during viral spread, will inform future studies aimed at defining the molecular processes regulating successful HIV-1 replication in both infected and susceptible target T cells and the associated immunological dysfunction causing AIDS. In an era in which ex vivo manipulation of T cells is increasingly deployed for diverse immunotherapy strategies, our findings have clear importance beyond the sphere of HIV-1 and define concepts of T cell activation that could be considered in future immunomodulatory strategies.

## Experimental Procedures

### Cell Culture

Jurkat T cell lines and HeLa TZM-bl and 293T cells were cultured, infected, or transduced as described in Supplemental Experimental Procedures. Primary CD4 T cells were isolated from peripheral blood of healthy donors by Ficoll gradient centrifugation and negative selection (Miltenyi Biotec), cultured, and infected as described in Supplemental Experimental Procedures.

### Virus Production and Infection

HIV-1 was prepared from the molecular clone pNL4.3 (NIH AIDS Reagent and Reference Program [ARRP]) by transfecting 293T cells using Fugene 6 (Promega) and infectious virus titered on HeLa TZM-bl cells using Bright-Glo Luciferase assay (Promega). Jurkat cells or primary CD4 T cells were infected with HIV-1 NL4.3 by spinoculation (Groppelli et al., 2015) and incubated for 48 hr prior to use. Alternatively, cells were infected with VSV-G-pseudotyped virus generated by transfecting 293T cells with pNL4.3, pNL4.3 ΔNef, or pNL4.3 ΔEnv and pMDG (Naldini et al., 1996). Infection was quantified by flow cytometry staining for intracellular Gag (Groppelli et al., 2015).

### Quantitative Phosphoproteomics and Mass Spectrometry Analysis

Triple SILAC was performed on Jurkat CE6-1 cells and incorporation confirmed by MS. Jurkat cells labeled with heavy (R10K8) amino acids were infected with HIV-1 and mixed with uninfected target Jurkat cells labeled with light (R0K0) amino acids. Medium-labeled (R6K4) cells were used as an internal reference. Infected and uninfected T cells were mixed and incubated for 0, 5, 20, or 40 min prior to lysis. All time-point samples were buffer exchanged, reduced, and alkylated and subsequently digested using filter aided sample preparation (FASP). Digested peptides were fractionated via hydrophilic interaction chromatography (HILIC) and enriched for phosphopeptides using titanium immobilized metal affinity chromatography (Ti-IMAC) and analyzed by high-resolution nano-liquid chromatography electrospray ionization (nano-LC ESI) MS/MS. Raw MS data were processed using MaxQuant for protein assignment, quantification of peptides, phosphorylation, and phosphosite localization. Refer to Supplemental Experimental Procedures for more information.

### SDS-PAGE and Western Blotting

Cell lysates were prepared as described for MS. Proteins from an equal number of cells were separated by SDS-PAGE, transferred to nitrocellulose, and subjected to western blotting for total and phosphorylated proteins as described in Supplemental Experimental Procedures. Blots are representative of two or three independent experiments. Densitometry quantification of bands was performed using ImageJ. The band intensity for each phosphoprotein was normalized to the corresponding total protein at each time point and plotted as the mean fold change in protein phosphorylation (where t = 0 min was normalized to 1) from multiple experiments.

### HIV-1 Replication Assays

Jurkat T cells were infected with VSV-G-pseudotyped HIV-1. Forty-eight hr post-infection, viral supernatants were harvested and Gagp24 quantified by ELISA (Jolly et al., 2007b). Virion infectivity was determined by luciferase assay using HeLa TZM-bl reporter cells. HIV-1 cell-cell spread was measured by quantitative real-time PCR (Jolly et al., 2007b, Martin et al., 2010), and data are shown as the fold increase in HIV-1 DNA compared to the albumin housekeeping gene and normalized to baseline (0 hr), reflecting de novo reverse transcription in newly infected target T cells during cell-cell spread. Alternatively, Jurkat 1G5 target T cells containing a luciferase reporter gene driven by the HIV-1 long terminal repeat (LTR) were used and cell-cell spread measured by luminescence assay (Groppelli et al., 2015).

### Immunofluorescence Microscopy

Quantification of T cell-T cell contacts and VS was performed as described previously (Jolly et al., 2004). Conjugates were defined as two closely apposed cells consisting of one HIV-1-infected T cell and one target T cell. VSs were defined as conjugates showing enrichment of HIV-1 Env and Gag to the site of cell-cell contact (Jolly et al., 2004, Rudnicka et al., 2009). Images were acquired using the DeltaVision ELITE Image Restoration Microscope (Applied Precision) coupled to an inverted Olympus IX71 microscope and a CoolSNAP HQ2 camera, deconvolved with softWoRx 5.0 and processed using Huygens Professional v4.0 and Adobe Photoshop C3. Quantification of fluorescence intensity was performed using ImageJ. A region of interest at the contact site (ROI 1) was selected and compared to a region on the opposite side of the cell (ROI 2). The integrated density was adjusted for the size of region of interest and for background, and the fold enrichment of fluorescence signal at the contact zone was determined for at least 20 contacts from two independent experiments.

### Statistical Analysis

Statistical significance was calculated using the Student’s t test or the Mann-Whitney test. For multiple comparisons, statistical significance was calculated using the parametric ANOVA test with Bonferroni correction. Significance was assumed when p < 0.05. All tests were carried out in GraphPad Prism 6 software.

## Author Contributions

C.J. conceived the study. A.C.L.L. designed, performed, and analyzed the quantitative phosphoproteomics and western blotting experiments. S.S. performed the viral replication experiments and analyzed the data. M.S. performed the TCR reconstitution experiments and western blotting and analyzed the data. C.J. and A.C.L.L. wrote the paper, with contributions from all authors.

## Accession Numbers

The mass spectrometry proteomics data have been deposited to the ProteomeXchange Consortium via the PRIDE partner repository with the dataset identifier PRIDE: PD005658.

## Supplemental Information

Document S1. Supplemental Experimental Procedures and Figures S1–S5

Table S1. All Identifications from Both Phosphoproteome and Proteome Duplicate Experiments

Table S2. Curated Qualitative and Quantitative Data for All Four Time Points from Both Phoshoproteome and Proteome Single and Duplicate Experiments

Table S3. Comparison of Overlapping and Unique Phosphoproteins Identified in HIV-Positive Donor T Cells and Target T Cells

Table S4. Comparison TCR Signaling Proteins Identified in Our Study and Previous Dynamic Phosphoproteomic Studies Using Direct Antibody-Based TCR Stimulation

Table S5. Comparison of Phosphorylation Changes Identified in Target T Cells in This Study during Cell-Cell Spread Compared to Cell-free Infection

Document S2. Article plus Supplemental Information
