# Supplementary material for: HIV-1 Activates T Cell Signaling Independently of Antigen to Drive Viral Spread
Source: Cell Rep. 2017 Jan 24;18(4):1062–74. doi: 10.1016/j.celrep.2016.12.057 (PMC5289937; doi:10.1016/j.celrep.2016.12.057)
Supplement: Document S1. Supplemental Experimental Procedures and Figures S1–S5 [file mmc1.pdf]

Cell Reports, Volume *18*

Supplemental Information

HIV-1 Activates T Cell Signaling Independently of Antigen to Drive Viral Spread

Alice C.L. Len, Shimona Starling, Maitreyi Shivkumar, and Clare Jolly

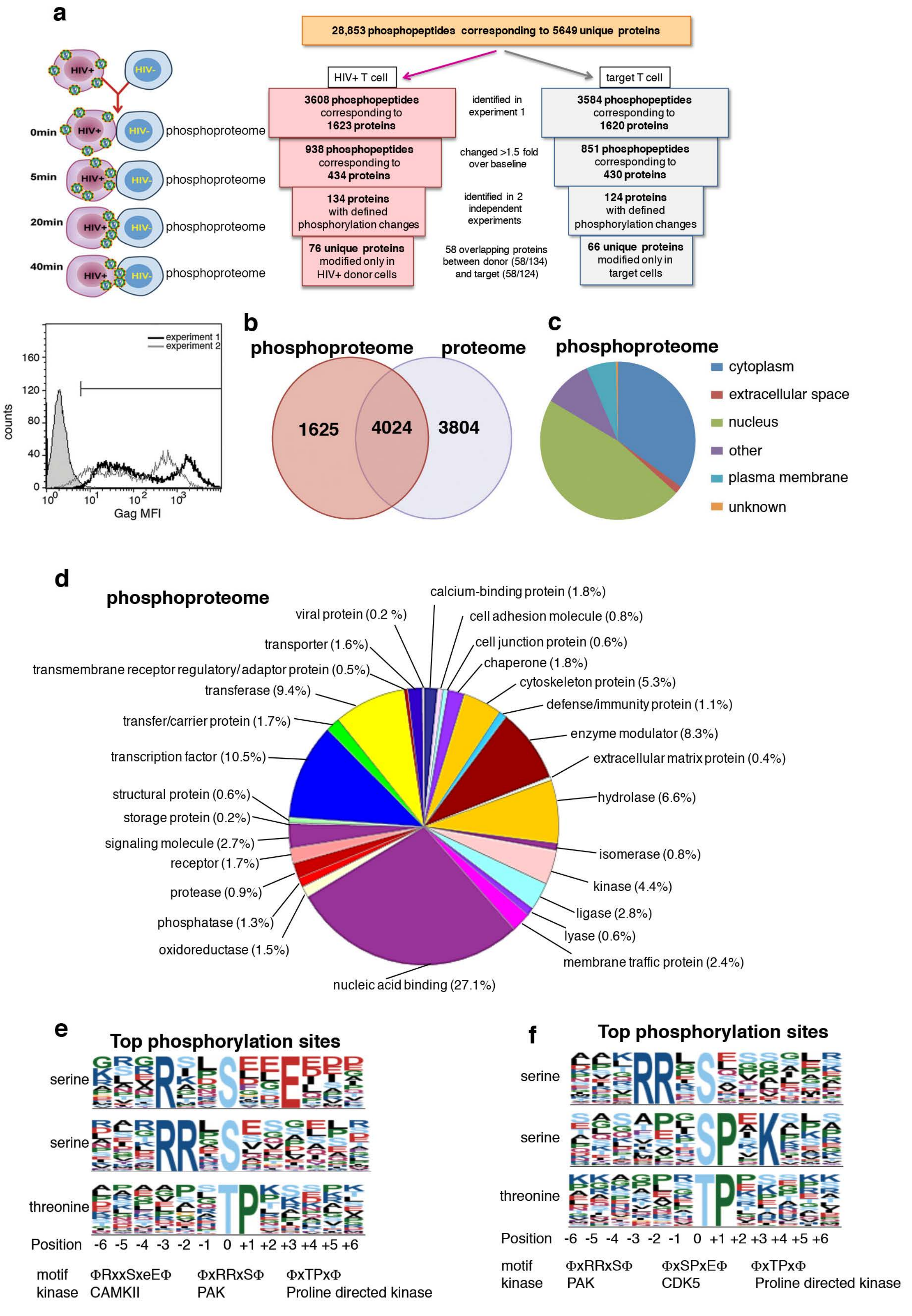

Figure S1 related to Figure 1. Phosphoproteomics screen with bioinformatics analysis of coverage. A SILAC labeled HIV-1 infected and uninfected Jurkat T cells were mixed and incubated for different periods of time before being lysed and analyzed by mass spectrometry. The number of proteins with mapped phosphorylation site changes (differentially expressed >1.5 fold over time, 0/5 min, 20/5 min and 40/5 min) in HIV-1 infected donor T cells and target T cells identified in experiment 1 and confirmed in experiment 2. Inset: Flow cytometry quantification of HIV-1 infection for experiment 1 (black line) and 2 (grey line) compared to uninfected cells (filled) measured by intracellular Gag staining. **B** Overlap between proteins identified in the total proteome and phosphoproteome analysis. **C** Subcellular localization of proteins identified in the phosphoproteome analysis combined for HIV-1 infected donor T cells and target T cells. **D** Geneontology analysis (PANTHER) of protein function for phosphoproteome analysis combined for HIV-1 infected donor and target T cells. **E and F Kinase motif analysis**. Top putative kinase motifs and kinases identified using Motif-x to have a change in phosphorylation over 40 minutes in HIV-1 infected donor T cells (E) and uninfected target T cells (F).

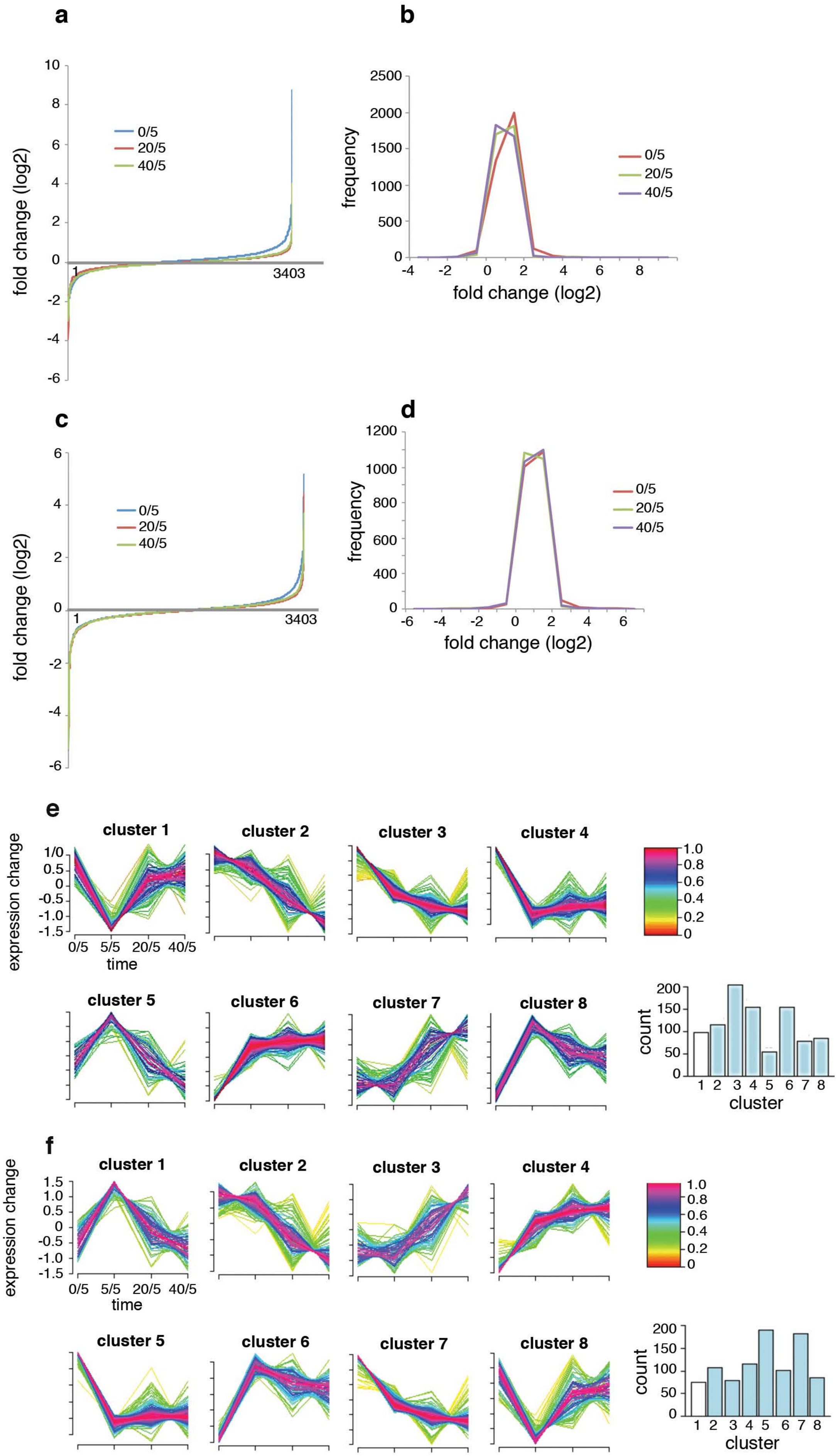

Figure S2 related to Figure 1. Distribution of phosphopeptides in HIV-1 infected T cells and uninfected target cells. A and C Distribution of phosphopeptides identified in HIV-1+ donor **(A)** and target cell **(C)** samples in experiment 1 based on fold change (log2) for all non-redundant phosphopeptides identified. **B and D**. Frequency distributions of 0 min/5 min, 20 min/5 min and 40 min/5 min SILAC fold change (log2) in the HIV-1+ donor **(B)** and target cells **(D)** sample. **E and F GProX cluster analysis depicting distinct groups of kinetically similar responsive proteins. E** HIV-1 infected T cells. **F** Uninfected target cells. Phosphopeptides showing a >2 fold change in phosphorylation over 40 min were subjected to unsupervised clustering with the Fuzzy c-means algorithm. Eight distinct patterns of dynamic changes could be classified, whereby the number of proteins included in each cluster is indicated. Color-coded membership represents how well a single protein pattern fits with the general profile of the cluster.

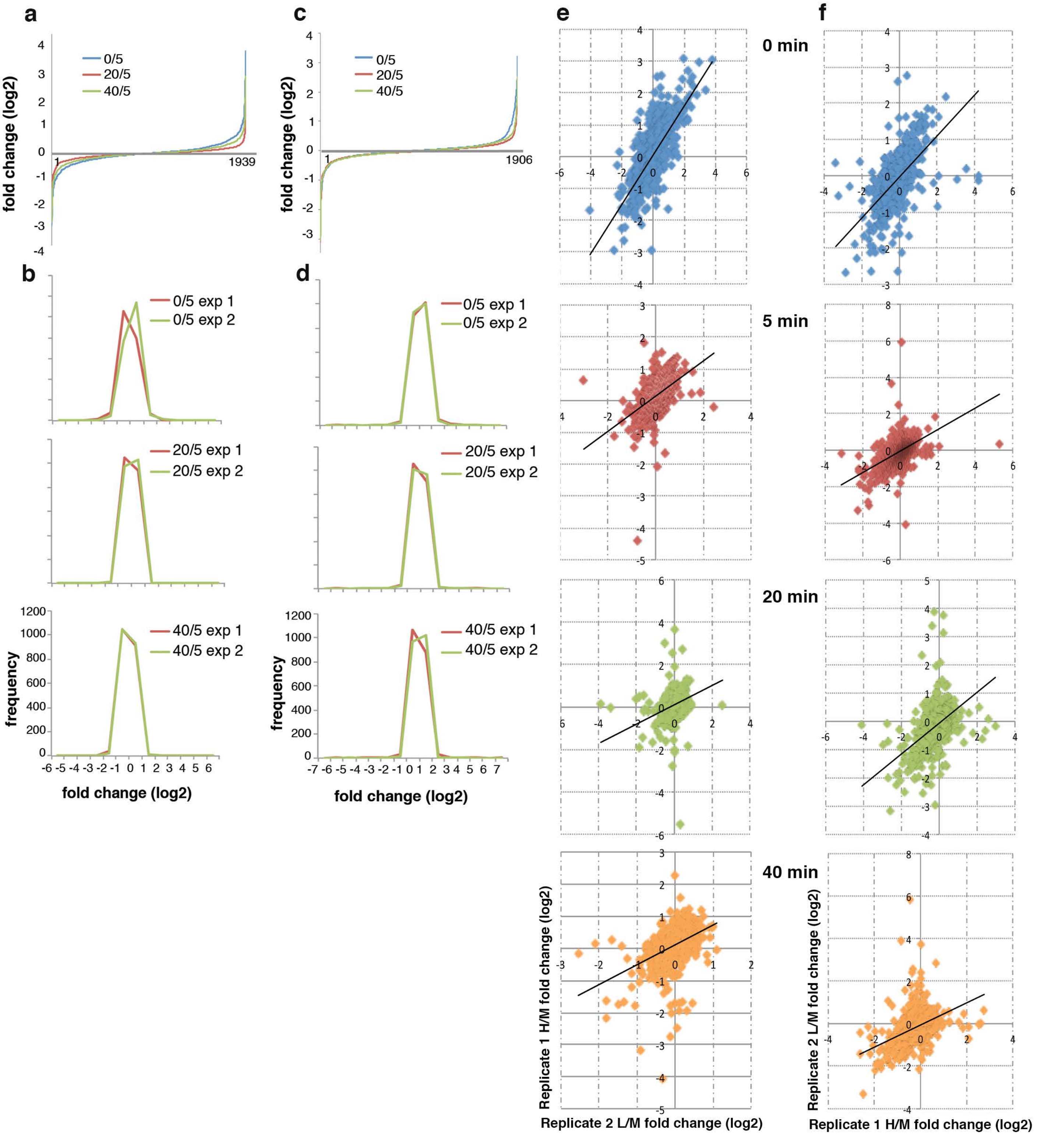

Figure S3 related to Figure 1. Replicate experiments show reproducible phosphopeptide distribution. Distribution of phosphopeptides identified in the HIV-1+ donor cells **(A and B)** and the uninfected target T cells **(C and D)** based on their average fold change (log2) for all non-redundant phosphopeptides identified. Refer to legend in Figure S2. **E and F Measurement of SILAC reproducibility between replicate experiments. E** HIV-1+ donor cells. **F** Uninfected target T cells. For each time point, the y-axis indicates the relative phosphopeptide abundance measured in experiment 1 and the x-axis experiment 2.

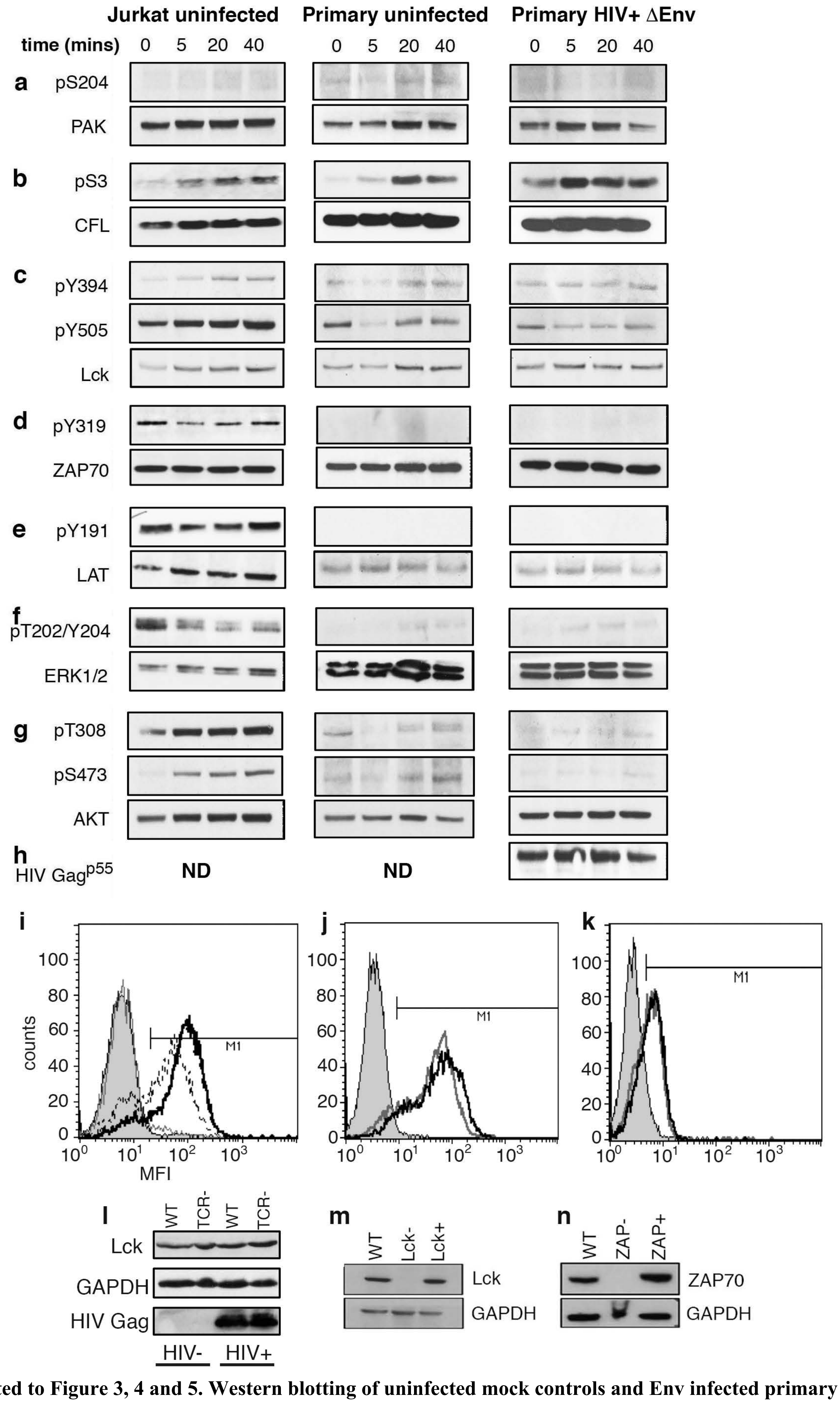

Figure S4 related to Figure 3, 4 and 5. Western blotting of uninfected mock controls and Env infected primary T cells. Protein phosphorylation was determined by western blotting as described for Fig. 3. Left panel = uninfected Jurkat T cells incubated with uninfected Jurkat target cells. Middle panel = uninfected primary CD4 T cells incubated with uninfected primary CD4 target T cells. Right panel = primary CD4 T cells infected with VSV-G pseudotyped Env HIV-1 and incubated with uninfected primary CD4 target T cells. ND = not done. **I-N Validation of Jurkat mutant cell lines and reconstitution.** I Flow cytometry analysis of TCR plasma membrane expression on Jurkat T cells. Unstained Jurkat T cells (grey filled), TCR-negative J.RT3-T3.5 Jurkat cells (grey line), WT TCR+ve Jurkat cells (black dotted line) and TCR-reconstituted J.RT3-T3.5 Jurkat cells (black solid line). **J** HIV infection does not downregulate cell surface TCR expression on Jurkat T cells. Unstained Jurkat T cells (grey filled), uninfected Jurkat T cells (grey line) and HIV-1 infected Jurkat T cells (black line). **K** HIV infection does not downregulate cell surface CD3 expression on Jurkat T cells. Unstained Jurkat T cells (grey filled), uninfected Jurkat T cells (grey line) and HIV-1 infected Jurkat T cells (black line). **L** Western blotting confirming that TCR-negative J.RT3-T3.5 Jurkat cells retain expression of Lck and that HIV-1 infection does not modulate Lck levels. M-N Western blotting confirming the protein expression phenotype of Lck-negative **(M)** and ZAP70-negative **(N)** Jurkat T cells and reconstituted cell lines.

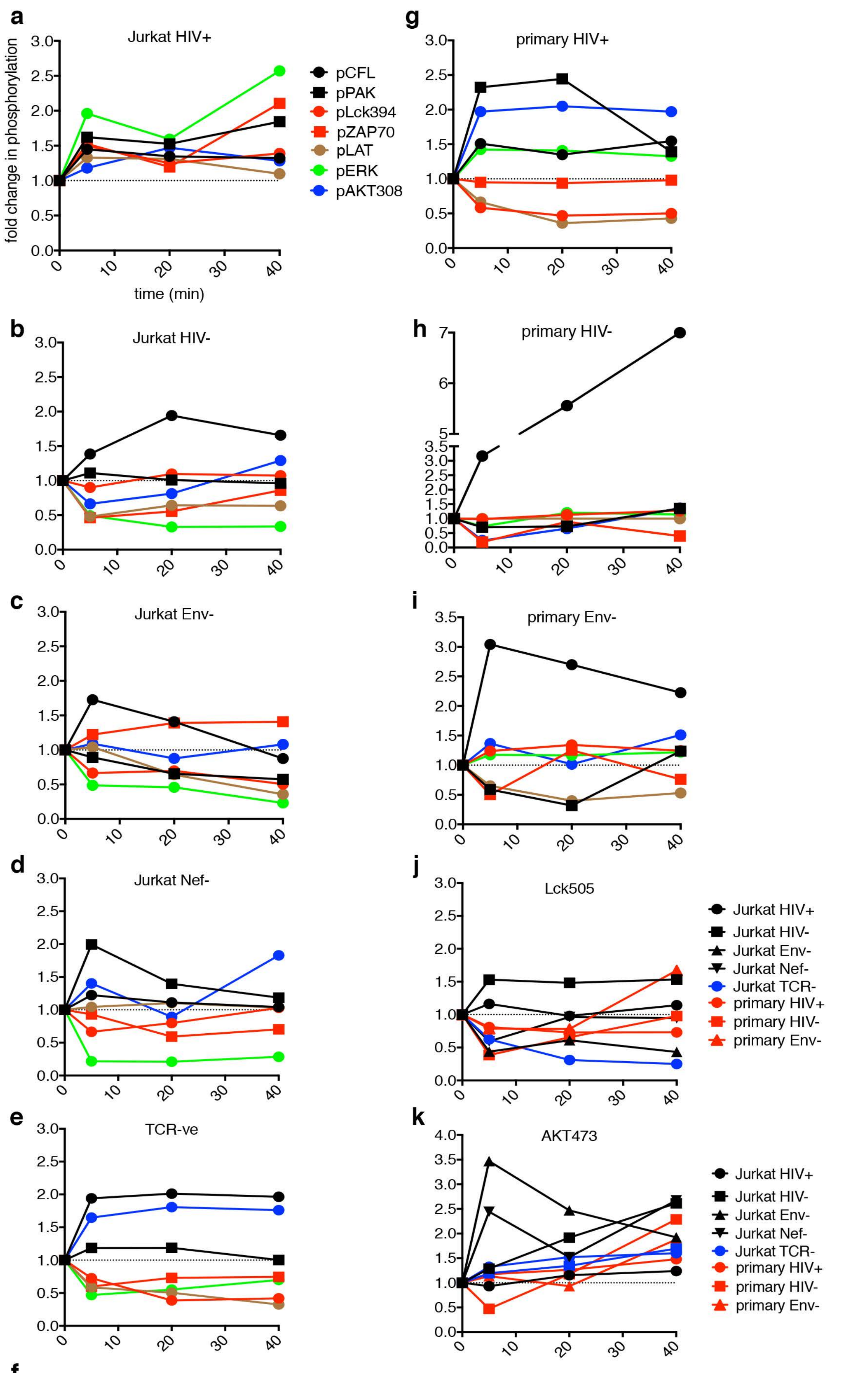

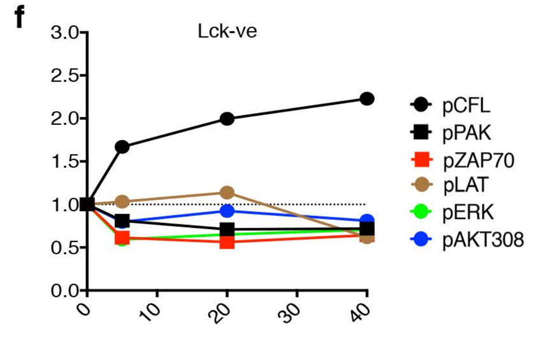

Figure S5 related to Figure 3, 4, 5 and S4. Densitometry quantification of western blots. Densitometry quantification of western blots was performed using Image J. The band intensity for each phosphoprotein was determined and normalized to the corresponding total protein for each time point for two or three independent experiments. Data are plotted as the mean fold change in protein phosphorylation over time where t=0min was normalized to 1. To aid visualization, phosphoproteins are plotted for each condition **(A-I)**, with the exception of Lck505 (the inhibitory Lck site that remained unchanged) and AKT473 (activation was HIV infection independent) that are shown on separate graphs **(J and K)**.

Supplemental Tables.

Table S1 related to Figures 1-4. All identifications from both phosphoproteome and proteome duplicate experiments.

Table S2 related to Figures 1-4. Curated qualitative and quantitative data for all 4 time points from both phoshoproteome and proteome single and duplicate experiments.

Table S3 related to Figures 1-4. Comparison of overlapping and unique phosphoproteins identified in HIV+ donor T cells and target T cells.

Table S4 related to Figures 1 and 2. Comparison TCR signaling proteins (KEGG) identified in our study and previous dynamic phosphoproteomic studies using direct antibody-based TCR stimulation.

Table S5 related to Figure 1. Comparison of phosphorylation changes identified in target T cells in this study compared to Wojcechowskyj et al., 2013.

Supplemental Experimental Procedures

Cell Culture

Jurkat T cell lines clones E6-1 (American Type Culture Collection [ATCC] TIB-152); Lck-negative JCAM1.6 (Straus and Weiss, 1992); Lck-reconstituted JCAM1.6 (a gift from A. Weiss, UCSF, USA); ZAP70-negative JP116 (Williams et al., 1998); ZAP70-reconstituted JP116 (Williams et al., 1998); TCR/CD3 negative J.RT3-T3.5 (Ohashi et al., 1985) and LFA-1 negative β2.7 (Weber et al., 1997) were grown in RPMI 1640 medium (Invitrogen) supplemented with 10% (v/v) heat inactivated fetal calf serum (FCS) penicillin/streptomycin (100 U mL^{-1}, 100 μg mL^{-1}). J.RT3-T3.5 cells were reconstituted with TCR alpha and beta chain expression by transducing cells with lentivirus prepared by co-transfecting 293T cells with the TCR expression plasmid (a gift from H. Stauss and E. Morris, UCL), Gag-pol packaging plasmid and VSV-G (Naldini et al., 1996). Primary CD4 T cells were isolated from peripheral blood of healthy donors by Ficoll gradient centrifugation and negative selection (Miltenyi Biotec). For virus infection, PBMC were activated in 5μg/ml PHA and 10IU IL2 for 3 days and then cultured in RPMI 20% FCS (v/v) with 10IU IL2. For immunofluorescence, primary CD4 target T cells were prepared without activation. HeLa TZM-bl cells (Center for AIDS Reagents, National Institutes of Biological Standard and Control, UK (CFAR, NIBSC)) and HEK 293T cells (ATCC) and were maintained in Dulbecco's Modified Eagle Medium (DMEM) supplemented with streptomycin, penicillin and 10% FCS.

SDS-PAGE and Western blotting

Cell lysates were prepared as described for MS. Proteins from an equal number of cells were separated by SDS-PAGE, transferred onto nitrocellulose and blocked in PBS with 0.05% Tween 20 (v/v) and 5% BSA (w/v). The following antibodies against phosphorylated and total proteins were used: $pLck^{Y394}$, $pLck^{Y505}$ and total Lck (Cell Signaling Technology, CST); $pZAP70^{Y319}$ and total ZAP70 (CST and New England Biolabs); $pLAT^{Y191}$ and total LAT (CST); $pPAK1^{S204}$ and total PAK1 (Assaybiotech and New England Biolabs) $pCFL^{S3}$ and total CFL (CST); $pERK1/2^{T202/Y204}$ and total ERK1/2 (CST); $pAKT^{T308}$, $pAKT^{S473}$ and total AKT (CST, Signalway Antibody and Biolegend) and HIV-1 Gag p55/p24 (CFAR, NIBSC, UK). HIV-1 Nef antiserum was a gift from E. Potton (UCL). Primary antibody was detected using polyclonal goat anti-rabbit or anti-mouse HRP (Dako) and chemiluminescence was detected using ECL (GE Healthcare). Alternatively, blots were blocked overnight in PBS with and 5% skim milk (w/v), incubated with primary antibodies followed by goat anti-rabbit IRdye 800CW infrared secondary antibodies and imaged using an Odyssey Infrared Imager (LI-COR Biosciences). Blots are representative of two or three independent experiments. Densitometry quantification of bands was performed using Image J. The band intensity for each phosphoprotein was normalized to the corresponding total protein at each time point and plotted as the mean fold change in protein phosphorylation (where t=0min was normalized to 1) from multiple experiments.

Quantitative phosphosphoproteomics and mass spectrometry

<u>SILAC labeling</u>

SILAC was performed on Jurkat CE6-1 using one of three labels (Silantes, Munich, Germany) in RPMI devoid of arginine and lysine (Thermo Fisher Scientific): 'light' label L-arginine and L-lysine (R0K0); 'medium' label L-arginine-$^{13}C_6$-$^{14}N_4$ and L-lysine-$^{2}H_4$ (R6K4); or 'heavy' label L-arginine-$^{13}C_6$-$^{15}N_4$ and L-lysine-$^{13}C_6$-$^{15}N_4$ (R10K8) supplemented with penicillin/streptomycin (100 U mL^{-1}, 100 μg mL^{-1}), 10% (v/v) in house dialyzed FCS using 3.5 kDa cut off dialysis membrane (Spectra/Por), 2mM GlutaMAX™-I (Gibco/Life technologies), 1 x MEM Vitamin Solution

(Gibco/Life technologies), 1 x Insulin-Transferrin-Selenium (Gibco/Life technologies) and 1mM Sodium pyruvate (Sigma). For replicate SILAC experiments identical low passage cells maintained in non SILAC RPMI were washed and transferred using one of three SILAC labeled media and grown as described above for at least 6 doublings. Initial seeding of 2 x 10^5 cells mL^{-1} in SILAC labeled media was used and upon reaching 3 doublings the cells were split down to 2 x 10^5 cells mL^{-1} and cultivated for a further 3 doublings in SILAC labeled media.

Preparation of cell lysates from HIV-1 infected and uninfected T cell co-culture

To avoid label bias two replicate experiments where a light and heavy label swap was conducted with the HIV-1 infected donor and target (uninfected) T cells. Dynamic phosphorylation changes upon conjugate formation between donor and target cells over 4 time points, 0, 5, 20 and 40 min were analyzed. To enable the comparison across all 4 time points for either donor or target cells for each experiment, both cell populations were independently labeled with the medium label, mixed for each time point and the samples were subsequently pooled together to make an internal reference and an equal amount spiked into each light/heavy VS time points. A large number of cells are required for a two tiered proteome and phosphoproteome analysis. A total of 2.4 x10^7 cells from 6 technical replicates for each light and heavy label per time point and 3 technical replicates for the donor/target labeled with medium label were all carried out in 6 well plates and combined based on cell count with the ratio of 1(light):1(medium pool):1(heavy) to give a total of 7.2 x 10^7 cells per time point. To do this, 6 well plates were coated with 0.0025% poly-L-lysine and 4 x10^6 target T cells were added to each coated well to form a monolayer of cells for 2h at 37°C. Donor cells (4 x10^6 per well) were added to target T cell and briefly centrifuged for <30 sec to synchronize conjugates and incubated at 37°C for 5, 20 and 40 min. For 0 min time point donor cells were additionally treated with protease and phosphatase inhibitor cocktails cOmplete ULTRA Tablets (Roche) and Phosstop (Roche), respectively, for 15 min prior to being mixed with target cells and harvested immediately. At the end of each time point the supernatant was removed and each technical replicate was lysed with 1mL 4% (w/v) SDS, 0.1M Tris-HCl pH 7.6 and 0.1M DTT supplemented with the aforementioned protease and phosphatase inhibitors. Each sample was probe sonicated on ice to shear genomic content with 4 bursts of 15 sec with 60 sec rest on ice between each burst using amplitude of 20%. The samples were then centrifuged at 12,000 x *g* for 20 min and technical replicates for each time point combined. For each replicate experiment the medium labeled samples of all technical replicates from each time point were combined and subsequently all time point samples were further combined to create a pooled internal standard that was added to each light/heavy labeled time point.

FASP

Filter Aided Sample Preparation (FASP) was performed as previously described (Wisniewski et al., 2009). Briefly, each of the time point samples (9mL) were heated at 95°C for 5 min then diluted with 60 ml UA buffer (100 mM Tris-HCL, pH 8.5, 8 M urea) and subsequently loaded onto 4 individual Nanosep, 10K Omega spin filters in 300 µL lots (PALL). Spin filters were centrifuged at 8000 x *g* repeatedly until all the supernatant from the different samples were filtered through. The filters were then washed three times with UA buffer, and proteins were alkylated by the addition of 150 µL of 50 mM iodoacetamide (Sigma). Filters were then washed 5 times with UA buffer and proteins digested twice with sequencing-grade modified trypsin (Promega). Digestion with trypsin was carried out at 30°C in 100 µL of 50mM NH_4HCO_3 to a final trypsin concentration of 0.18 µg μL^{-1} overnight and then for a further 6h in the same conditions. Filters were then washed with 500 mM NaCl and resulting peptides acidified with trifluoroacetic acid (TFA) in preparation for desalting.

HILIC

Eluted peptides were desalted using a Sep-Pak SPE C18 column (Waters) according to manufacturer's instructions. The desalted peptides were dissolved in 80% (v/v) CH_3CN with 0.1% (v/v) TFA and subsequently fractionated into 24 fractions using a Hydrophilic Interaction Liquid Chromatography (HILIC) column (TSKgel Amide-80,4.6*250mm, 5µm [Tosoh Biosciences, Germany]) on a Dionex HPLC (Ultimate 3000) system. The flow rate applied was 0.6mL min^{-1}, varying concentrations of Buffer A: 0.1% (v/v) TFA in 18 milliQ H_2O and Buffer B: 0.1% (v/v) TFA in CH_3CN, were used to separate the peptides using the following gradient: 80%-80% B in 20 min, and then 80%B -70%B in 10 min, and then 70%B -60%B in 30 min. Each fraction was split in a 1:19 ratio for subsequent proteome and phosphoproteome analysis, respectively, and dried using a centrifugal evaporator (SpeedVac, ThermoSavant, Thermo Scientific) prior to mass spectrometry analysis.

Phosphopeptide enrichment using Ti IMAC beads

Phosphopeptides were batch enriched from each peptide fraction using 100µL of 10 mg mL^{-1} slurry of MagReSyn titanium immobilized metal affinity chromatography (Ti-IMAC) magnetic microspheres (ReSyn Biosciences Ltd, South Africa). The microspheres were firstly washed with 80µL 0.5% (v/v) NH_4OH then washed 3 times with 200µL 80% (v/v) CH_3CN with 1% (v/v) TFA. This was followed by 3 washes with 200µL of 1M glycolic acid, 80% (v/v) CH_3CN, 5% (v/v) TFA. Each wash was for 2 min with shaking prior to magnetic separation to separate the microspheres from the liquid and the resulting liquid was discarded. Each sample was reconstituted with 100µL of a 1 M glycolic, acid 80% (v/v) CH_3CN, 5% (v/v) TFA. The HILIC fractionated peptide samples and microspheres were then combined and shaken for 20 min at room temperature. The microspheres were then initially washed with 200µL of 1 M glycolic, 80%

(v/v) CH_3CN, 5% (v/v) TFA followed by a further 3 washes of 200µL 80% (v/v) CH_3CN, 1% (v/v) TFA. As previously, washes were incubated for 2 min with shaking prior to magnetic separation and the liquid was discarded. The microspheres were then incubated for 2 min with 80µL of 0.5% (v/v) NH_4OH and after magnetic separation the liquid was transferred to a tube containing 60µL of 10% (v/v) CH_2O_2. This was repeated a further 2 times pooling all 3 elutions for each sample. The samples were then dried as above using a SpeedVac.

LC ESI MS/MS Analysis

Trypsin-digested peptides, either enriched for phosphopeptides through Ti IMAC or not, were separated using an Ultimate 3000 RSLC (Thermo Scientific) nanoflow LC system. On average 0.5 µg was loaded with a constant flow of 5 µL mL^{-1} onto an Acclaim PepMap100 nanoViper C18 trap column (100 µm inner-diameter, 2cm; Thermo Scientific). After trap enrichment, peptides were eluted onto an EASY-Spray PepMap RSLC nanoViper, C18, 2 µm, 100 Å column (75 µm, 50 cm; ThermoScientific) with a linear gradient of 2–40% solvent B (80% [v/v] CH_3CN with 0.08% [v/v] CH_2O_2) over 124 min with a constant flow of 300 nL min^{-1}. The HPLC system was coupled to a linear ion trap Orbitrap hybrid mass spectrometer (LTQ-Orbitrap Velos Pro, Thermo Scientific) via a nano-electrospray ion source (Thermo Scientific). The spray voltage was set to 1.8 kV, and the temperature of the heated capillary was set to 250 °C. Full-scan MS survey spectra (*m/z* 335–1800) in profile mode were acquired in the Orbitrap with a resolution of 60,000 after accumulation of 1,000,000 ions. The fifteen most intense peptide ions from the preview scan in the Orbitrap were fragmented by collision-induced dissociation (normalized collision energy, 35%; activation Q, 0.250; and activation time, 10 ms) in the LTQ after the accumulation of 10,000 ions. Maximal filling times were 500 ms for the full scans and 100 ms for the MS/MS scans. Precursor ion charge state screening was enabled, and all unassigned charge states as well as singly charged species were rejected. The lock mass option (445.120024) was enabled for survey scans to improve mass accuracy (Olsen et al., 2005). For the phosphorylated samples MultiStage Activation (MSA) was enabled (neutral loss mass list – 24.49, 32.66, 48.99, 97.98). Data were acquired using the Xcalibur software.

Quantification and bioinformatics analysis

The raw mass spectrometric data files obtained for each experiment were collated into a single quantitated data set using MaxQuant (version 1.3.0.5) (Cox and Mann, 2008) and the Andromeda search engine software (Cox et al., 2011). Enzyme specificity was set to that of trypsin, allowing for cleavage N-terminal to proline residues and between aspartic acid and proline residues. Other parameters used were: (i) variable modifications, methionine oxidation, protein N-acetylation, gln → pyro-glu, and Phospho (STY) – specifically for the phosphorylation site enrichment samples; (ii) fixed modifications, cysteine carbamidomethylation; (iii) database: target-decoy human MaxQuant (UniprotSwall-human, downloaded 20141203); (iv) heavy labels: R6K4 and R10K8; (v) MS/MS tolerance: FTMS- 10ppm, ITMS- 0.6 Da; (vi) minimum peptide length, 7; (vii) maximum missed cleavages, 2; (viii) maximum number of labeled amino acids, 3; (ix) false discovery rate, 1%; and (x) minimum number of peptides, 2. Peptide ratios were calculated for each arginine- and/or lysine-containing peptide as the peak area of labeled arginine/lysine divided by the peak area of non-labeled arginine/lysine for each single-scan mass spectrum. Peptide ratios for all arginine- and lysine-containing peptides sequenced for each protein were averaged. Data were normalized using 1/median ratio value for each identified protein group per labelled sample. Corrected phospho peptide ratios were calculated by dividing the phospho-peptide ratio by the equivalent normalised standard experiment protein ratio. Network analysis was done through the use of QIAGEN's Ingenuity®Pathway Analysis (IPA®, QIAGEN Redwood City, www.qiagen.com/ingenuity) and top pathways represented using Gephi graph and network analysis software (Bastian et al., 2009). Canonical pathways analysis identified the pathways from the Ingenuity Pathways Analysis library of canonical pathways that were most significant to the data set. Molecules from the data set that met the cut-off of >1.5-fold and were associated with a canonical pathway in Ingenuity's Knowledge Base were considered for the analysis. The significance of the association between the data set and the canonical pathway was measured in two ways: (1) A ratio of the number of molecules from the data set that map to the pathway divided by the total number of molecules that map to the canonical pathway is displayed; (2) Fisher's exact test was used to calculate a *p*-value determining the probability that the association between the genes in the data set and the canonical pathway is explained by chance alone. We used Gephi (https://gephi.org/users/publications/) to graphically represent the molecular relationship between molecules that were generated from IPA. Molecules are represented as nodes, and the biological relationship between two nodes is represented as an edge (line). All edges are supported by at least one reference from the literature or from canonical information stored in the Ingenuity Pathways Knowledge Base. Human, mouse, and rat orthologs of a gene are stored as separate objects in the Ingenuity Pathways Knowledge Base, but they are represented as a single node in the network.

Supplemental References

Bastian, M., Heymann, S., and M., J. (2009). Gephi: an open source software for exploring and manipulating networks. In International AAAI Conference on Weblogs and Scoal Media.

Cox, J., and Mann, M. (2008). MaxQuant enables high peptide identification rates, individualized p.p.b.-range mass accuracies and proteome-wide protein quantification. Nat Biotechnol *26*, 1367-1372.

Cox, J., Neuhauser, N., Michalski, A., Scheltema, R.A., Olsen, J.V., and Mann, M. (2011). Andromeda: a peptide search engine integrated into the MaxQuant environment. J Proteome Res *10*, 1794-1805.

Ohashi, P.S., Mak, T.W., Van den Elsen, P., Yanagi, Y., Yoshikai, Y., Calman, A.F., Terhorst, C., Stobo, J.D., and Weiss, A. (1985). Reconstitution of an active surface T3/T-cell antigen receptor by DNA transfer. Nature *316*, 606-609.

Weber, K.S., York, M.R., Springer, T.A., and Klickstein, L.B. (1997). Characterization of lymphocyte function-associated antigen 1 (LFA-1)-deficient T cell lines: the alphaL and beta2 subunits are interdependent for cell surface expression. J Immunol *158*, 273-279.

Williams, B.L., Schreiber, K.L., Zhang, W., Wange, R.L., Samelson, L.E., Leibson, P.J., and Abraham, R.T. (1998). Genetic evidence for differential coupling of Syk family kinases to the T-cell receptor: reconstitution studies in a ZAP-70-deficient Jurkat T-cell line. Mol Cell Biol *18*, 1388-1399.

Wisniewski, J.R., Zougman, A., and Mann, M. (2009). Combination of FASP and StageTip-based fractionation allows in-depth analysis of the hippocampal membrane proteome. J Proteome Res *8*, 5674-5678.
